# Mapping large bodies of research in environmental sciences: insights from compiling evidence on the recovery and reuse of nutrients found in human excreta and domestic wastewater

**DOI:** 10.1186/s13750-025-00366-5

**Published:** 2025-07-14

**Authors:** Robin Harder

**Affiliations:** 1 Environmental Engineering Group, Department of Energy and Technology, Swedish University of Agricultural Sciences (SLU), Uppsala, Sweden; 2 Architecture, Design and Civil Engineering, Zurich University of Applied Sciences (ZHAW), Winterthur, Switzerland

**Keywords:** Literature review, Systematic map, Evidence synthesis

## Abstract

Mapping evidence on a particular research topic among others aims to provide a comprehensive overview of the topic along with a searchable database of relevant literature. When attempting to map large bodies of research, mappers may soon find themselves in a situation where the resources available for the mapping are incommensurate to the number of studies to be handled. This typically requires either a narrower scope of the map or a streamlined mapping process. Grounded in a comparison of five evidence maps on the topic of recovery and reuse of nutrients found in human excreta and domestic wastewater—some of them systematic, some not—the present paper sets out to quantify the potential effect of procedural differences on mapping outcomes. Ultimately, the goal is to discern the factors that matter most for comprehensive and balanced mapping outcomes. This exploration suggests that a good search strategy is key when mapping large bodies of research, especially so when terminology is barely standardized. The paper also sheds light to an issue that could be described as differential search term sensitivity and specificity (compound search terms that are not equally sensitive and specific across all subdomains of the map) and that may deserve more attention in evidence mapping. Drawing from my experiences from compiling the online evidence platform Egestabase, the paper sketches how this issue might be mitigated. In addition, the paper outlines several measures that can help achieve substantial efficiency gains, and offers reflections on how to set priorities and navigate tradeoffs when a standard systematic mapping process appears not to be viable and not strictly necessary.

**Supplementary Information:**

The online version contains supplementary material available at 10.1186/s13750-025-00366-5.

## Background

In many research fields, publication rates are increasing from year to year. Consequently, research synthesis gains in importance. One important step in research synthesis is to identify and catalogue relevant literature. Systematic mapping [12] is one way to achieve this and typically provides an overview of the distribution and abundance of evidence. Researchers trained in evidence synthesis generally are familiar with pertinent systematic map guidelines and standards [5], while this is not necessarily the case for researchers trained as domain experts in the research topics to be mapped. Moreover, for domain experts, mapping typically serves as a basis for further research synthesis rather than being the end goal. Therefore, they may want the mapping process to be as streamlined as possible.

Myself, I have actively contributed for almost a decade to synthesizing research on the recovery and reuse of nutrients found in human excreta—including in domestic wastewater and treatment residues such as sewage sludge and sewage sludge ash. In a comprehensive literature review, together with colleagues, I attempted to articulate and summarize options to recover nutrients found in human excreta for reuse in agriculture [11]. Roughly around the same time, other researchers attempted to systematically map evidence on the recovery and reuse of nutrients found in domestic wastewater [8, 13]. We only learnt of each others’ work after it got published. Once I compared the studies included in the two evidence bases, I was surprised by the low overlap–many studies featured in one evidence base were not featured in the other and vice versa. In fact, even after correcting for differences in scope and time period, still only about a tenth of the studies included in at least one of the two evidence bases was included in both. I got curious, we joined forces, and roughly a year later we had managed to secure funding to expand our previous work by means of a systematic map on the recovery and reuse of nutrients found in human excreta and domestic wastewater [14, 15]—and to build the online evidence platform Egestabase (www.egestabase.net) that catalogues this evidence [10]. In parallel, I got the opportunity to help building an evidence base for nutrient recovery from human urine that underpinned an assessment of knowledge evolution in this field [1]. My perspective on evidence mapping thus is that of a seasoned domain expert with limited formal training in evidence synthesis.

Systematic mapping is appealing in terms of the rigor of the process and the reproducibility of the results. However, throughout the process of compiling Egestabase—which involved screening of over 150,000 and coding of over 15,000 studies—it soon became evident that the overwhelming number of studies to be reviewed threatened to bog down the mapping process to such an extent that resources would likely run out before the mapping gets completed. The challenge thus was to find ways to streamline the process, while still ensuring that the outcomes are valid and guidelines and standards are followed as best as possible.

Ultimately, this paper wishes to offer reflections on how to achieve efficiency gains, what to prioritize, and how to navigate tradeoffs when the resources available for the mapping are incommensurate to the overwhelming number of studies to be handled—and when it is deemed acceptable to streamline the mapping process (a systematic mapping process may in fact not always be strictly necessary). For the paper to be more tangible, it is grounded in a comparison of the evidence bases mentioned above—similar in scope but with surprisingly different outcomes—and sets out to quantify how procedural differences may have affected mapping outcomes.

## Overview of previous evidence bases

**Fig. 1:**
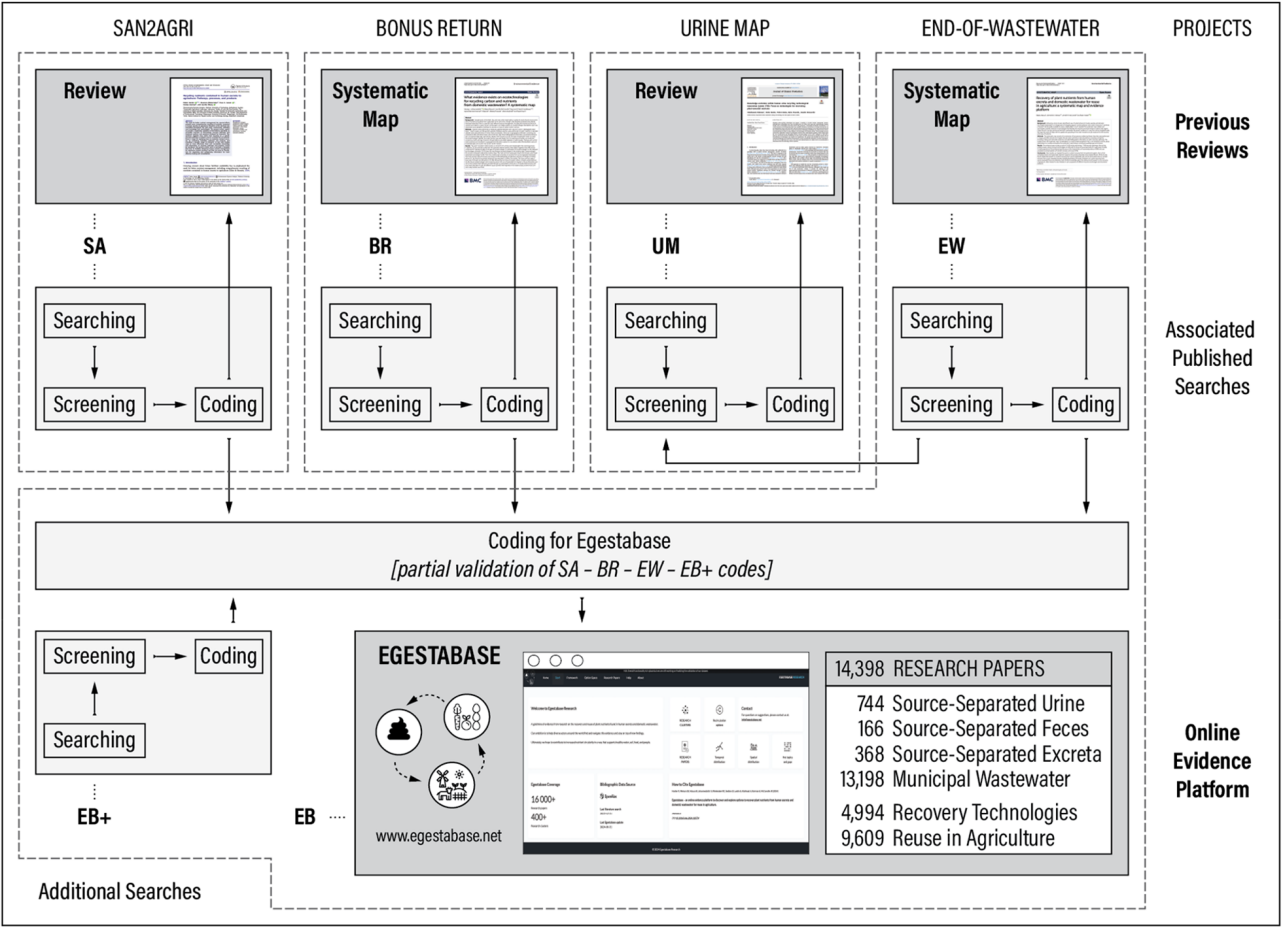
Interrelations between the four previous reviews and how they formed the basis for the Egestabase online evidence platform

The present paper is based on five evidence bases. Four of them stem from previous reviews (Table 1) that are partly interrelated and contributed to developing and populating the fifth—the online evidence platform Egestabase (Fig. 1).

**Table 1:** List of previous reviews that contributed to developing and populating Egestabase

Code	Type	Title and context	Citation(s)
SA	Review	Recycling nutrients contained in human excreta to agriculture: pathways, processes, and products > *Conducted as part of a postdoctoral project on recycling organic matter and nutrients from sanitation to farming systems to regenerate soil and land (SAN2AGRI)*	[11]
BR	Systematic map	What evidence exists on ecotechnologies for recycling carbon and nutrients from domestic wastewater? A systematic map > *Conducted as part of a multinational BONUS project on reducing emissions by turning nutrients and carbon into benefits (BONUS RETURN)*	[8, 13]
UM	Review	Knowledge evolution within human urine recycling technological innovation system (TIS): Focus on technologies for recovering plant-essential nutrients > *Conducted as part of a doctoral project on sustainability assessment of nutrient recycling systems from wastewater with a particular focus on urine (URINE MAP)*	[1]
EW	Systematic map	Recovery of plant nutrients from human excreta and domestic wastewater for reuse in agriculture: a systematic map and evidence platform > *Conducted as part of a collaborative project on the recovery and reuse of plant nutrients in human excreta and domestic wastewater (END-OF-WASTEWATER)*	[14, 15]

### Description of evidence bases

#### Goal

Four of the evidence bases (BR, UM, EW, EB) were compiled with the goal of identifying all the evidence, while the SA evidence base was compiled with the goal of identifying distinct options for nutrient recovery and reuse (and thus not necessarily all the evidence per option).

#### Eligibility

Eligibility criteria (Table S1.1 in OSM 1) appear to be largely similar overall (Table 2). *Population.* The UM evidence base covers only human urine while the other four (SA, BR, EW, EB) cover domestic and municipal wastewater more broadly—including fractions such as human urine and feces, as well as residues such as sewage effluents or sludges. *Interventions* and *outcomes*. All evidence bases cover the recovery and/or reuse of plant nutrients, possibly along with organic carbon. The BR evidence base in addition also covers the recovery and/or reuse of organic carbon without concurrent recovery of nutrients. *Study types*. All evidence bases cover primary research. The EB evidence base in addition also considers secondary research.

**Table 2:** Overview of eligibility criteria applied in the previous reviews and Egestabase

Evidence base	Populations(s)	Intervention(s)	Outcomes(s)	Study type(s)
BR	Domestic and municipal wastewater (including fractions and residues)	Carbon and nutrient recirculation (recovery and reuse)	Nutrients and carbon	Primary research
SA	Domestic and municipal wastewater (including fractions and residues)	Nutrient recirculation (recovery and reuse)	Nutrients	Primary research
UM	Human urine	Nutrient recirculation (recovery and reuse)	Nutrients	Primary research
EW	Domestic and municipal wastewater (including fractions and residues)	Nutrient recirculation (recovery and reuse)	Nutrients	Primary research
EB	Domestic and municipal wastewater (including fractions and residues)	Nutrient recirculation (recovery and reuse)	Nutrients	Primary and secondary research

#### Searching

Search strings (Table S1.2 in OSM 1) followed a similar pattern across previous reviews—namely a combination of population, intervention, and outcome terms in the form of ‘<population terms> AND <intervention terms> AND <outcome terms>’. Each term can be associated with a concept (Table S1.3 in OSM 1). For example, the population terms ‘urine’ and ‘yellowwater’ (including the variations ‘yellow water’ and ‘yellow-water’) represent the concept ‘human urine’; similarly, the intervention terms ‘recycle’ (including the variations ‘recycled’ and ‘recycling’), ‘circulate’ and ‘recirculate’ (both also including variations) together represent the concept ‘recycle’. Concepts searched for in previous reviews are listed in Table 3.

**Table 3:** Concepts searched for in previous reviews per search string element

Search string element	Concepts
Population	sanitation, human urine, human feces, human excreta, domestic and municipal wastewater
Intervention	treatment, recovery, recycling, reuse, agriculture
Outcome	nutrient, nutrient-rich product

Not all concepts and search terms were used in all previous reviews (Table 1.4 in OSM 1). Moreover, when compiling Egestabase, a set of additional searches was also performed (EB+)—these searches aimed specifically at expanding the coverage of individual subdomains of the map (e.g., ‘urine AND struvite precipitation’, ‘feces AND vermicomposting’, ‘blackwater AND pasteurization’). While these searches were used to populate Egestabase, they were not a part of the EW review (which was based on a single compound search string as per the systematic map protocol). Finally, search periods and databases searched differed across searches (Table 4).

**Table 4:** Search period and databases searched in previous reviews

Search	Period	Database(s)
BR	2013–2017	Scopus, Web of Science Core Collection, Google Scholar, eThOS Electronic Theses Online Service, DOAJ Directory of Open Access Journals, DART-Europe E-Theses Portal
SA	Until 2017	Scopus
UM	Until 2021	Scopus, Web of Science Core Collection
EW	Until 2022	Scopus, Web of Science Core Collection
EB+	Until 2023	Scopus

Note that no specific EB search is listed here as the EB evidence platform is the result of the BR, SA, UM, EW and EB+ searches

#### Screening

The bulk of the screening was performed by a single reviewer in four cases (SA, UM, EW, EB) and by a team of four reviewers in one case (BR). Two more differences are worth noting. In the BR review, it was decided that studies describing recovery technologies are to be considered only if an intended reuse of the recovered product is explicitly mentioned. In the SA review, only a subset of studies identified by the search were screened (more specifically, screening was stopped once it appeared likely that the remaining studies would not add any new options for nutrient recovery and reuse).

#### Coding

For three evidence bases (BR, EW, EB), the reviewed studies were coded along six coding dimensions; for two evidence bases (SA, UM), the reviewed literature was coded along a reduced set of coding dimensions (Table 5). Like for screening, the bulk of the coding was performed by a single reviewer in four cases (SA, UM, EW, EB) and by a team of four reviewers in one case (BR). The coding categories applied within each coding dimension are detailed in Table S1.5 in OSM 1 for each of the five evidence bases.

**Table 5:** Coding dimensions in previous reviews

Dimension	Description	BR	SA	UM	EW	EB
Topic	Study topic (e.g., recovery, reuse)	×	×	×	×	×
Source	Source stream (e.g., urine, blackwater)	×	×	×	×	×
Technology	Recovery technology (e.g., sorption, precipitation)	×	×	×	×	×
Target	Recovery target (e.g., nitrogen, phosphorus)	×	×		×	×
Product	Recovered product (e.g., struvite, biochar)	×	×		×	×
Reuse	Reuse of recovered product (e.g., crop fertilizer, animal feed)	×			×	×

#### Consistency checking

The BR and EW reviews were the only ones to report consistency checking. In the BR review, it consisted of parallel screening and coding of 24 full texts (1.8% of retrieved full texts) by multiple reviewers, followed by a discussion of disagreements. In the EW review, it involved a comparison of screening and coding outcomes with those of the BR review, as well as parallel screening of 1127 records (0.85% of deduplicated search results) and parallel coding of 89 records by multiple reviewers. The SA and UM reviews did not report consistency checking but nevertheless involved some level of ‘on the fly’ consistency checking for coding decisions (e.g., sifting through studies in a given coding category to see if there are any studies that do not fit there). Similar ‘on the fly’ consistency checking was also performed when assembling the EB online evidence platform—here the focus was mainly on harmonizing coding decisions for coding categories that are hard to tell apart and thus easily misclassified (e.g., struvite precipitation from sewage sludge liquid fractions versus struvite precipitation from sewage sludge ash leachate).

### Congruence of evidence bases

The congruence of evidence bases is best assessed for primary research from the period 2013 to 2017 that is indexed on Scopus or Web of Science—as all previous evidence bases have searched one or both bibliographic databases for this type of research and within this time period.

#### Overlap of studies

**Fig. 2:**
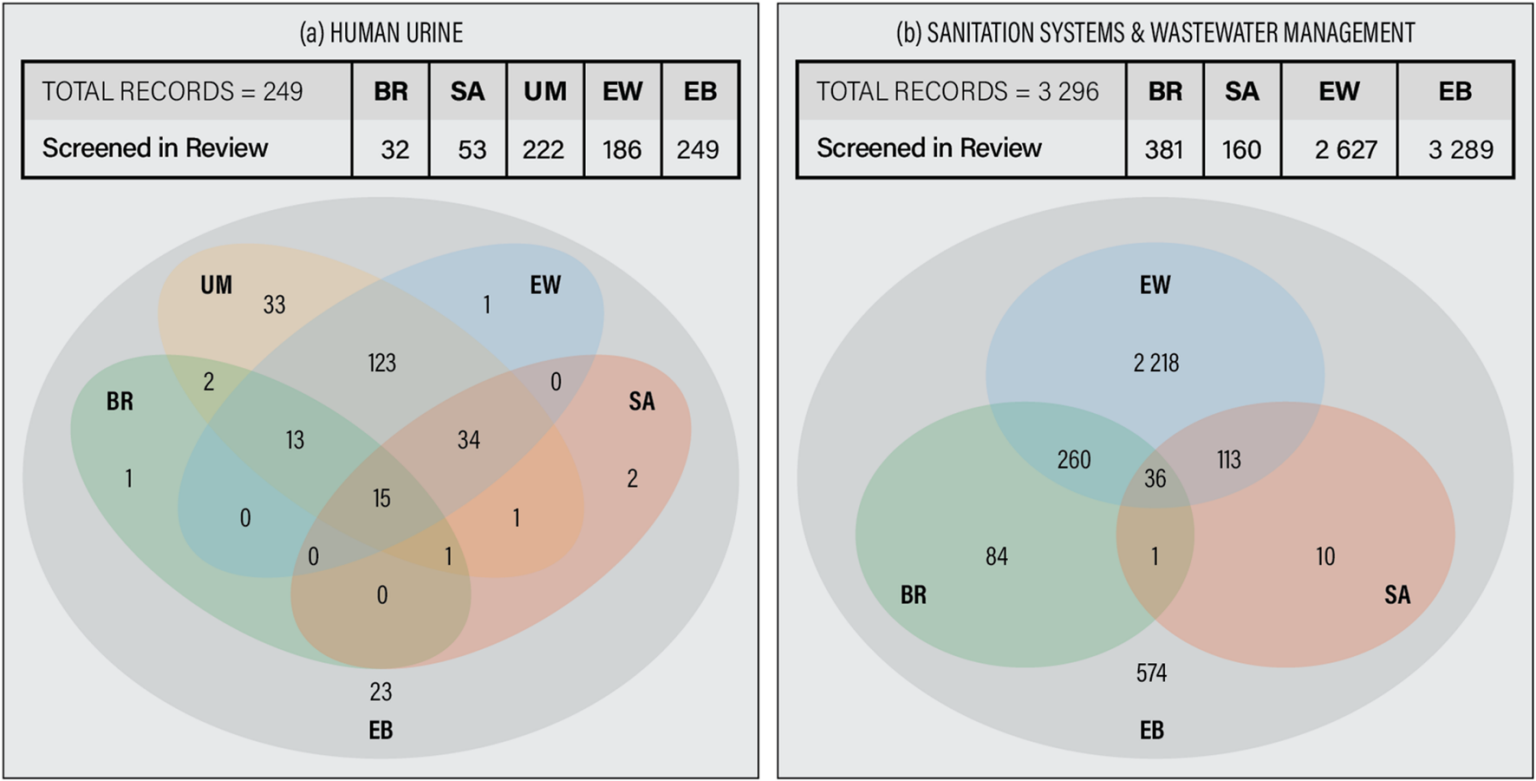
Overlap of evidence bases: comparison for **a** human urine and **b** sanitation systems and wastewater management more broadly (which includes human urine). Period 2013 to 2017. Numbers represent studies screened per evidence base that are included in at least one of the evidence bases

One way to assess the congruence of evidence bases is by assessing the overlap of studies. To this end, it appears expedient to compare studies that are included in at least one evidence base and that belong to a coding category that corresponds to nutrient recovery technologies or the reuse of recovered nutrients in agriculture. More specifically, for the UM evidence base this means that studies that broadly describe the concepts (rather than technologies) of source separation and urine diversion were not considered for the assessment of overlap. For the BR evidence base, studies on the recovery of carbon or energy without concurrent nutrient recovery were not considered. For the other three evidence bases (SA, EW, EB), all included studies were considered. For human urine as source stream, the assessment of overlap is based on 249 studies (Fig. 2a), for sanitation systems and wastewater management more broadly on 3296 studies (Fig. 2b).

The BR and SA evidence bases cover considerably fewer studies than the other evidence bases. At the same time, the EB evidence base covers a considerable number of studies not included in any of the other evidence bases. At first sight, it is rather surprising to not see more overlap across evidence bases given their rather similar eligibility criteria. On further consideration, it appears reasonable that the goal of mapping options rather than evidence in the SA review and the exclusion of studies without explicit mention of an intended reuse of the product in the BR review (both not explicitly reflected in the eligibility criteria) likely have an impact on the overlap of evidence bases.

#### Coverage across subdomains

**Fig. 3:**
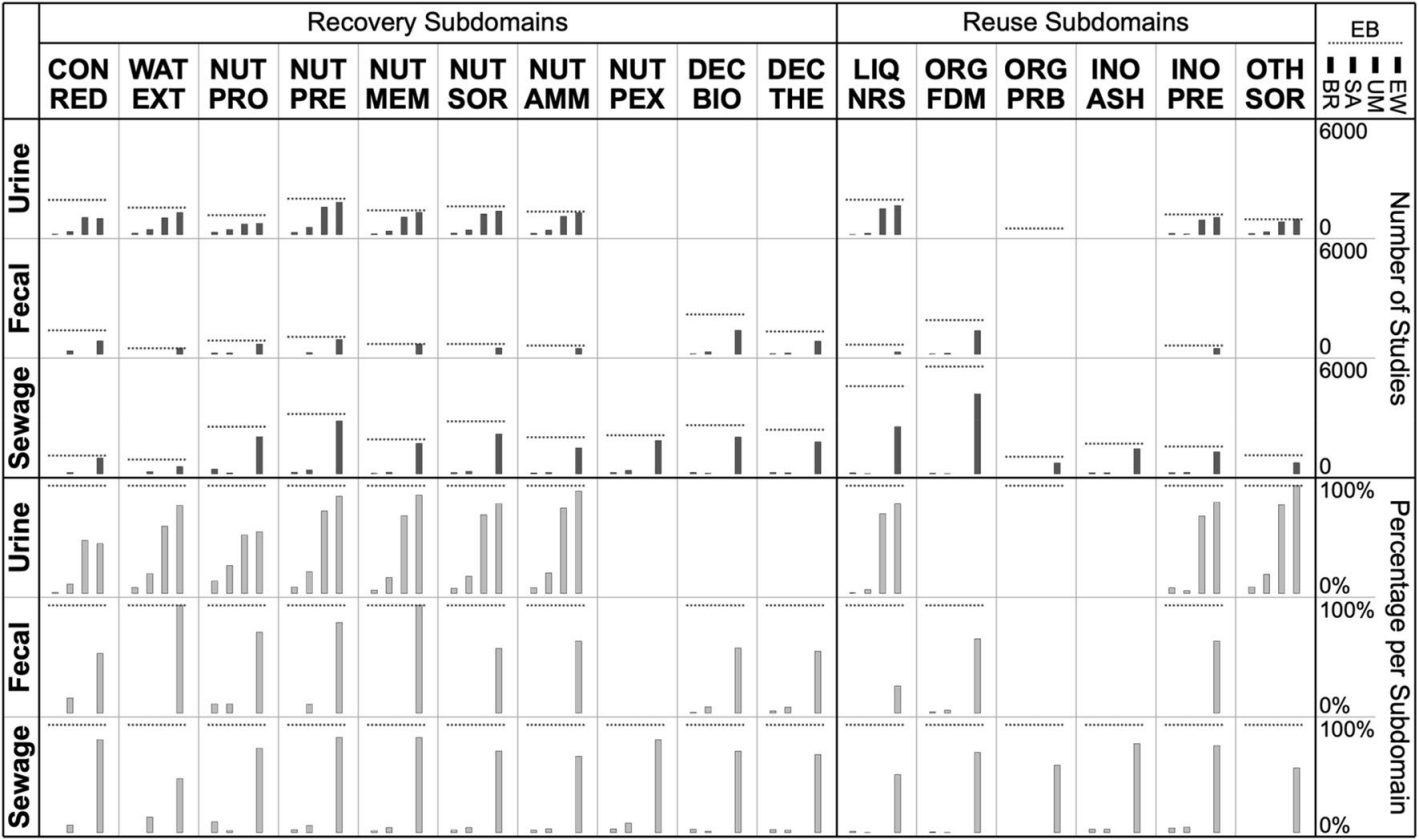
Distribution across subdomains of studies included in the EB evidence base (*dashed horizontal lines*) along with the fraction included in the other four evidence bases (*gray bars*). *Top* absolute numbers. *Bottom* percentages relative to EB coverage. Period 2013 to 2017

Another way to assess the congruence of evidence bases is by looking at the extent to which studies included in EB are included in the other evidence bases (BR, SA, UM, EW)—as EB is the evidence base with the largest coverage and all other evidence bases are subsets. This comparison becomes more interesting if done for distinct subdomains (Table 6) rather than the research domain as a whole. Unfortunately, the differences in coding schemes across evidence bases (notably in terms of coding categories) are such that not all coding categories neatly map onto the chosen subdomains (for instance, the technology categories ‘membrane filtration’ and ‘electrodialysis’ of the BR review can map onto either ‘contaminant reduction’, ‘water extraction’, or ‘nutrient extraction by membrane separation’ as per Table 6). Therefore, studies included in each evidence base were assigned the corresponding EB coding categories (that map unambiguously onto the subdomains as per Table 6) in order to analyze the distribution of evidence per evidence base across subdomains (Fig. 3).

**Table 6:** List of subdomains used for further comparisons across evidence bases

Recovery subdomains	Recovery technologies
CON.RED	Contaminant reduction
WAT.EXT	Water extraction
NUT.PRO	Nutrient extraction by protein rich biomass growth
NUT.PRE	Nutrient extraction by precipitation
NUT.MEM	Nutrient extraction by membrane separation
NUT.SOR	Nutrient extraction by sorption (including ion exchange)
NUT.AMM	Nutrient extraction by ammonia release and capture
NUT.PEX	Nutrient extraction by phosphorus release and capture
DEC.BIO	Biological decomposition of organic matter
DEC.THE	Thermal or hydrothermal decomposition of organic matter

Reuse subdomains	Recovered products
LIQ.NRS	Nutrient rich solution
ORG.FDM	Fecal derived biomass
ORG.PRB	Protein rich biomass
INO.ASH	Ashes or slags
INO.PRE	Precipitate (monomineral or multimineral)
OTH.SOR	Nutrient enriched sorbent material

The selection of subdomains is somewhat arbitrary—here, a rather coarse division was chosen that represents an aggregated version of the EB coding scheme

Regarding nutrient recovery (recovery subdomains in Fig. 3), across most applicable subdomains, a large fraction of the studies included in EB are also included in the UM and EW evidence bases. The exceptions are contaminant reduction (CON.RED), microalgae growth (NUT.PRO), biological decomposition (DEC.BIO) and thermal decomposition (DEC.THE)—it is particularly in these categories where the additional searches (EB+) yielded extra studies not found by the other searches. It can also be seen that the studies included in the BR and SA evidence bases, for most subdomains, amount to a rather small fraction of the studies included in EB. Similar patterns emerge regarding the reuse of recovered products (reuse subdomains in Fig. 3). For most applicable subdomains, studies included in the UM and EW evidence bases amount to a large fraction of the studies included in EB, while studies included in the BR and SA evidence bases amount to a rather small fraction of the studies included in EB. The BR evidence base probably covers much fewer studies because studies that did not explicitly mention an intended reuse were not considered. The SA evidence base features fewer included studies simply because many potentially relevant studies were not screened (as the goal was to identify distinct options for nutrient recovery and reuse rather than mapping all evidence for each identified option).

### Suitability of evidence bases for quantitative comparison

In summary, the evidence bases appear to be mostly similar in terms of eligibility criteria and the general structure of the underlying search strings and coding schemes. Time periods and databases searched do vary slightly, while key differences exist in search terms and coding categories. Other noteworthy differences are the use of a stopping rule during screening (SA review) and the decision to only consider studies that explicitly mention an intended reuse of products (BR review). The procedural differences imply that the evidence bases are less congruent than what one may expect when looking solely at eligibility criteria.

The similarities in general scope and structure in combination with the procedural differences and the rather low congruence of outcomes makes these evidence bases very suitable for scrutinizing procedural differences, in principle. In practice, the existence of differences in eligibility criteria (minor) and coding categories (major) means that comparing screening and coding decisions is not exactly straightforward albeit possible.

## Quantifying the effect of procedural differences

Before quantifying the effect of procedural differences, it seems indicated to assess the robustness of the screening and coding procedures that produced each evidence base. This was done based on a pairwise comparison of evidence bases at the level of individual studies (OSM2). Expressed as fraction of studies included in the respective evidence base, screening and coding irregularities were estimated to be below 5% in all evidence bases. It thus seems very unlikely that screening and coding irregularities are the main culprit for the rather low congruence of evidence bases. Neither does it appear likely that the extent of screening and coding irregularities would preclude quantification of the effect of procedural differences.

### Interpretational ambiguity

There seem to be cases where there are no objectively right or wrong screening or coding decisions. Interpretational ambiguity may arise for instance when a study is so poorly written that it is pretty much impossible to conclusively figure out what it is about. Based on the evidence bases considered here, it is not possible to quantify the overall effect of interpretational ambiguity. What can be done is to estimate the effect of the decision to either include or exclude studies that implicitly state the reuse of recovered products. While all previous reviews took a clear stance on this decision, failing to do so could also give rise to interpretational ambiguity. Some reviewers may tend to exclude studies with implicit reuse—this is a valid decision that can be motivated with good arguments. Other reviewers may tend to include studies with implicit reuse of recovered products—this is an equally valid decision and can also be motivated with good arguments. A study on ammonia stripping from wastewater for instance might still be deemed relevant, even if the intention is to remove rather than recover nitrogen, or the reuse of the recovered ammonium sulphate is not explicitly stated (it is a very common nitrogen fertilizer after all).

As a rough proxy for interpretational ambiguity, studies that were included in and excluded from the BR evidence base (where studies that do not explicitly state an intended reuse are excluded) were compared with what would have been included based on EB screening and coding decisions (where studies with an implicit reuse in agriculture are included). To this end, the EB coding categories were mapped onto the studies screened in the BR review (Table 7). For some subdomains, there is no or very little effect, while for others—such as ammonia release and capture (NUT.AMM) or the use of ashes in agriculture (INO.ASH)—the effect is considerable (differences of up to a factor of 2). While this comparison does not quantify the overall effect of interpretational ambiguity potentially present in the evidence bases, it at least suggests that interpretational ambiguity can have an effect whose magnitude may vary across subdomains.

**Table 7:** Estimated effect of the decision to include or exclude studies with implicit reuse of recovered products on coverage across subdomains. Period 2013 to 2017

		CON RED	WAT EXT	NUT PRO	NUT PRE	NUT MEM	NUT SOR	NUT AMM	NUT PEX	DEC BIO	DEC THE
URI	BR	2	5	4	13	2	5	3			
	BR*	4	6	4	13	2	6	3			
FEC	BR	0	0	1	0	0	0	0	0	2	1
	BR*	1	0	1	0	0	0	0	0	4	1
SEW	BR	0	0	50	26	3	17	5	9	16	11
	BR*	0	0	61	42	7	21	10	15	17	16

BR: Studies included in the BR evidence base (explicit reuse only)

BR*: Studies screened in the BR review that would have been included in the EB evidence base (thus including implicit reuse)

### Methodological choices

Given that interpretational ambiguity likely has some effect on screening and coding outcomes (the magnitude of which cannot be fully quantified for the evidence bases considered here), it seems expedient to find a way to eliminate (as best as possible) the effect of interpretational ambiguity from the further analysis. This can be achieved by mapping the EB coding categories to all evidence bases and then do (most of) the comparisons based on the respective EB coding categories rather than the original coding categories used in each review (except the comparison involving grey literature that is based on the original BR coding categories).

#### Grey literature

Grey literature refers to information produced outside of traditional publishing and distribution channels and includes theses, reports, policy literature, working papers, newsletters, government documents, speeches, white papers, urban plans and the like. The BR evidence base is the only one that includes grey literature in addition to white literature (i.e., literature that is produced by traditional academic or commercial publishing systems and typically underwent peer-review) found in books and journals. Looking into the distribution and abundance of grey and white literature in the BR evidence base (Table S3.1 in OSM3) suggested that, for many BR coding categories, the inclusion of grey literature added only few additional studies (mostly theses), if any. Nevertheless, a few BR coding categories would not have been identified by the review if it was not for the inclusion of grey literature (again, mostly theses)—these are subdomains for which very little evidence was found (quite possibly due to a suboptimal search strategy).

#### Bibliographic databases

Two important bibliographic databases that have been around for many years are Elsevier Scopus [2] and Clarivate Web of Science [3]. A rather new alternative is the open access bibliographic database OpenAlex [16]. Of course, these are not the only bibliographic databases, and it is unlikely that all relevant studies would be indexed on them, individually or in combination (although the majority of at least the white literature should be indexed on these databases). For three evidence bases (BR, UW, EW), searches involved both Scopus and Web of Science (OpenAlex search functionality became available only after the reviews had been conducted). The EW evidence base in particular lends itself for estimating the potential effect of the choice of bibliographic database as it is the most comprehensive one conducted on both Scopus and Web of Science.

**Fig. 4:**
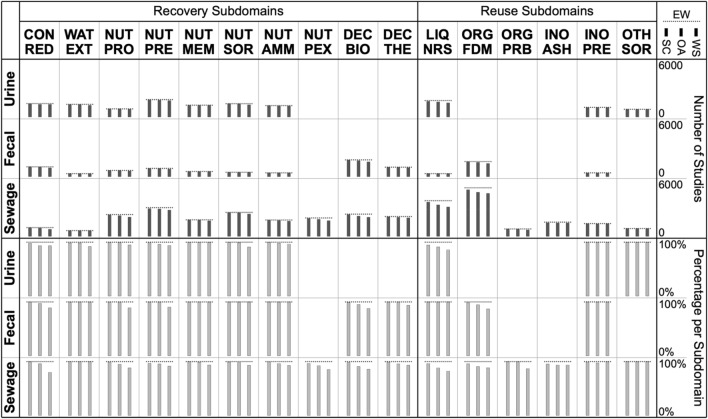
Distribution across subdomains of studies included in the EW evidence base (*dashed horizontal lines*) along with the fraction indexed on different bibliographic databases (*gray bars*). *Top figure* absolute numbers. *Bottom figure* percentages relative to EW coverage per category. *SC* Scopus, *WS* Web of Science, *OA* OpenAlex

Each study in the EW evidence base (found on Scopus and/or Web of Science) was matched against Scopus, Web of Science and OpenAlex (by DOI or alternatively article title) using their respective bibliographic API (Application Programming Interface). It would appear that, across the majority of subdomains, the majority of all studies are indexed on Scopus and OpenAlex, while a somewhat smaller number of studies is indexed on Web of Science (Fig. 4).

When attempting to compare bibliographic databases, one needs to distinguish what is being indexed from what is being retrieved through searches. This is important because the implementation of search functionality may vary across different bibliographic databases. Scopus for instance offers an ‘Article Title, Abstract, Keyword’ search (that presumably searches author keywords), whereas Web of Science offers a ‘Topic’ search that includes article title, abstract, author keywords and keywords plus (words or phrases that frequently appear in the titles of cited articles).

**Fig. 5:**
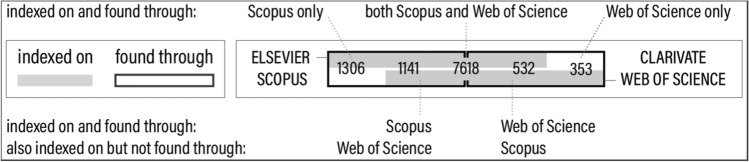
Overlap of Scopus and Web of Science in terms of hits (studies found by searching the respective database) and coverage (studies indexed on the respective database) for studies included in the EW evidence base

Interestingly, searches on Scopus yielded 1141 studies that are indexed on Web of Science but that were not found by searches on Web of Science; vice versa, searches on Web of Science yielded 532 studies that are indexed on Scopus but that were not found through searches on Scopus (Fig. 5). A possible explanation why this is the case remains elusive.

To conclude this section, it should be emphasized that the database overlap discussed here is highly domain specific and the comparison based solely on studies published in English. For other domains, when other languages are considered, or when other institutional subscriptions are used, a different picture may emerge.

#### Search terms

**Fig. 6:**
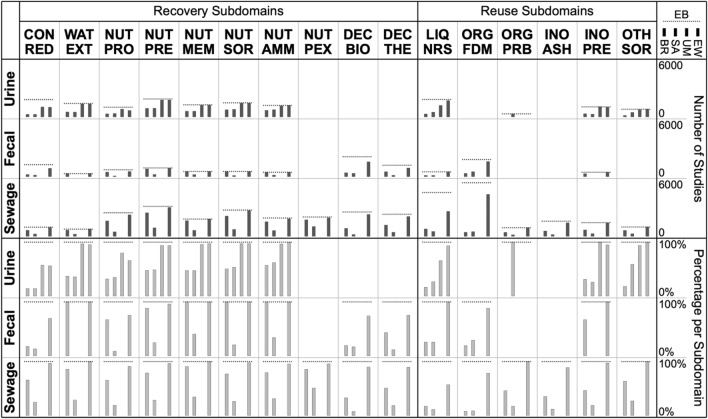
Distribution across subdomains of studies included in the EB evidence base (*dashed horizontal lines*) along with the fraction found by different search strings replicated based on previous review (*gray bars*). *Top figure* absolute numbers. *Bottom figure* percentages relative to EB coverage. Period: until 2023

The search terms that underpin the different evidence bases varied considerably (OSM 1). To assess the effect of search term selection, the respective search strings—as stated in the BR, SA, UM and EW reviews—were replicated and rerun on Scopus (this was necessary because not all studies reported search results). Compared to the studies included in the EB evidence base, the BR and SA search strings found a lower percentage of studies than the UM and EW search strings (Table 8). Mapping the EB coding categories to the search hits moreover suggested that the sensitivity of individual search strings varies considerably across subdomains (Fig. 6). Again, the findings presented in this section are highly domain specific.

**Table 8:** Performance of search strings stated in previous reviews in terms of search hits found that are included in EB

	Source-separated urine				Source-separated fecal matter				Domestic wastewater			
	Recovery		Reuse		Recovery		Reuse		Recovery		Reuse	
	#	%	#	%	#	%	#	%	#	%	#	%
BR	314	37	48	18	144	28	38	19	2780	67	1099	12
SA	323	38	75	29	80	16	55	28	997	24	891	9
UM	729	86	188	72	–	–	–	–	–	–	–	–
EW	722	85	244	94	389	76	174	88	3906	94	6738	71
EB	852	100	260	100	513	100	197	100	4168		9444	100

#### Constraining searches

When performing searches, it is possible to specify which search fields to search, and which search operators to use to connect multiple elements of the search string. Being more restrictive regarding search fields and search operators can be a way to reduce the number of search hits—ideally retaining as much as possible of the signal (i.e., studies one wants to find) while cutting as much as possible of the noise (i.e., studies one does not want to find). Another way to reduce the number of search hits is by adding additional inclusion or exclusion criteria—for instance by using additional search terms to be included or excluded.

**Fig. 7:**
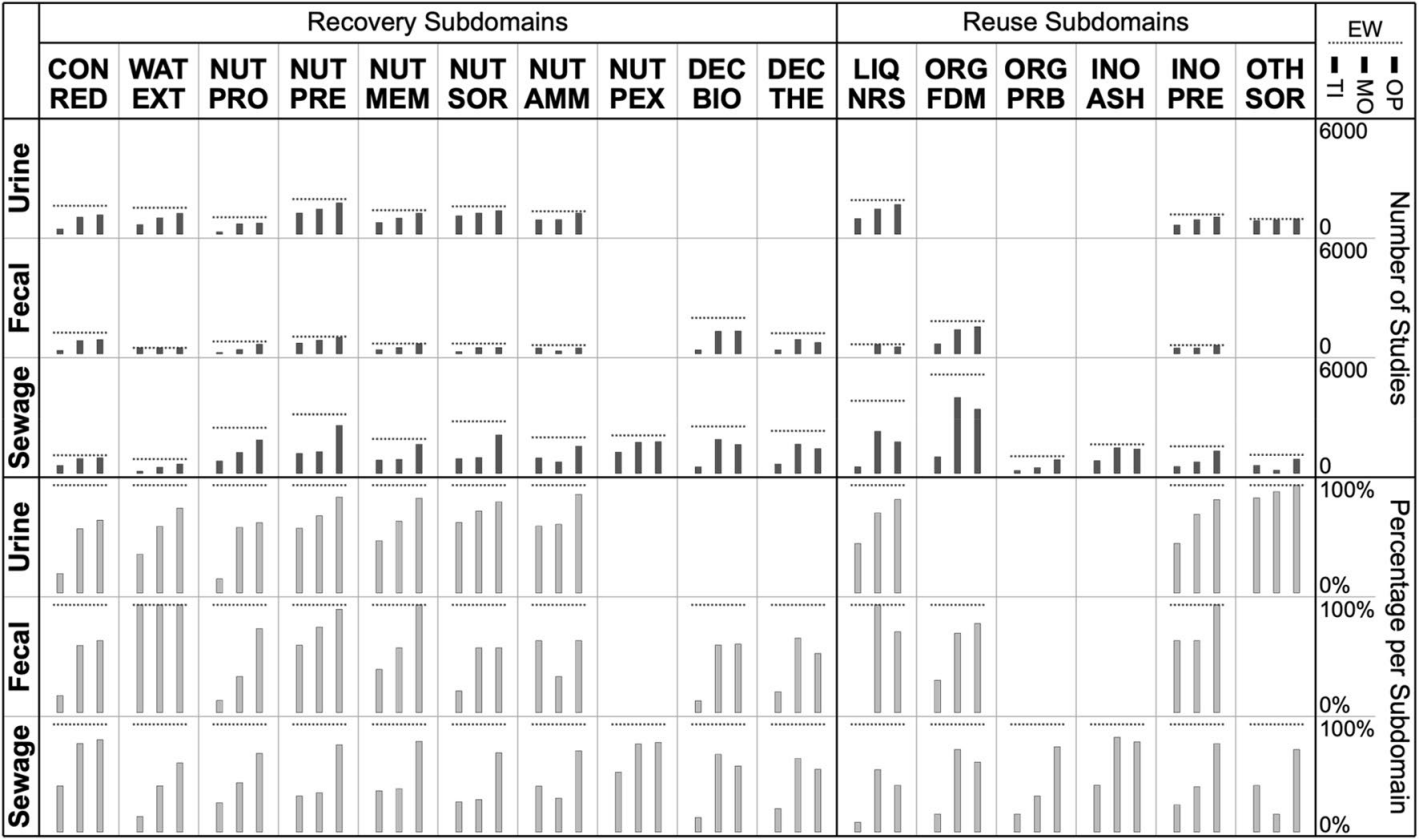
Distribution across subdomains of studies included in the EW evidence base (*dashed horizontal lines*) along with the fraction found through constrained searches (*gray bars*). *Top figure* absolute numbers. *Bottom figure* percentages relative to EW coverage. *EW* original EW search string. *TI* searches in only title field only. *MO* population term is constrained by adding terms such as ‘human’, ‘domestic’, ‘household’ and the like. *OP* intervention and outcome terms are connected by a proximity operator (W/3) rather than a simple boolean operator (AND). Period: until 2022

To assess the influence of various ways to constrain searches, the EW search was replicated and modified in three ways: searching only the title field (TI), constraining the population term (MO), and applying a more restrictive operator to join intervention and outcome terms (OP) (Table S3.2 in OSM3). The EB coding scheme was then mapped onto the respective search hits retrieved from Scopus (Fig. 7). For some subdomains, some or all of the more restrictive searches were almost as good as the original EW search, while for other subdomains, this was not the case. In other words, not all subdomains were equally affected by searching with more restrictive search settings.

### Relative effect of procedural choices

Across all evidence bases, the underlying screening and coding decisions appeared to be robust (in terms of screening and coding irregularities being estimated at under 5% as per OSM2). For the most part, the rather low congruence of evidence bases thus likely stems from procedural differences. While the effect of interpretational ambiguity could not be fully quantified, it appears reasonable to assume that it likely is less pronounced than the effect of methodological choices.

Regarding methodological choices, the inclusion of grey literature appeared to have the potential to expand the evidence base—not so much in quantitative terms but more so by potentially adding aspects for which no white literature was found (even if it may exist). In terms of what is indexed on the large bibliographic databases (i.e., Scopus, Web of Science, OpenAlex), there were some differences regarding the number of studies that can be potentially retrieved from each database and their distribution across subdomains (yet all subdomains were represented in all databases). More importantly and somewhat unexpectedly, in some cases the same search strategy implemented on one bibliographic database appeared to retrieve studies indexed on but not found through searching another bibliographic database. Overall, differences due to variations in bibliographic database coverage (indexation) appeared to be less pronounced than differences due to variations in search strings and their implementation. The choice of search terms seems to have the largest potential to skew mapping outcomes. Another aspect that may considerably skew mapping outcomes is to constrain searches to lesser search fields and use more restrictive search operators—in some cases one may get away with it, while in other cases one may miss out on a considerable number of relevant studies. It is important to emphasize at this point that the relative effect of procedural choices discussed in this section is a feature of both the evidence bases being compared and the knowledge domain they represent. In other words, the relative effect of procedural choices may be different for other knowledge domains or for a different set of evidence bases.

## Discussions and conclusions

To be clear upfront. Neither am I an expert in the field of evidence synthesis, nor do I wish to pretend to be one—my perspective is that of an expert on circular nutrient solutions. In this capacity, it is not my intention to replicate or question existing systematic mapping guidelines and standards. My contribution is to offer ideas and thoughts on how to navigate tradeoffs and set priorities when mapping large bodies of literature (notably when resources available for the mapping are incommensurate to the number of studies to be handled). This said, the reflections I articulate here are meant not as recommendations (this is what you should or should not do) but rather as suggestions (this is what you may want to consider).

### The challenge of mapping broad research fields

The sheer number of studies that exist within a broad research field like the recovery and reuse of nutrients found in human excreta and domestic wastewater make any mapping attempt a daunting task—unless of course the map focuses on a very specific and rather small subdomain of the research field. In this regard, James et al. [12] have emphasized the importance of finding an appropriate balance between sensitivity (search results contain all relevant studies) and specificity (low proportion of irrelevant studies in the search results)—and point out that a too sensitive but not so specific search can lead to a situation where search results are too extensive to screen within reasonable time and resource limits, while a too specific and not so sensitive search may miss vital evidence.

In hindsight, I think it is fair to say that the core search string that underpins Egestabase (EW search) was probably not specific enough—and thus meant that an overwhelming number of studies had to be screened and coded. It would appear that I was so eager to make Egestabase as comprehensive as only possible that I put up with way too many studies to be handled—frenziedly looking for ways how to plough through tens of thousands of studies as efficiently as possible. Probably this is because I had come across previous evidence maps that—as a seasoned domain expert—left me less than impressed regarding mapping outcomes (given that I quickly spotted substantial gaps and imbalances in the distribution of literature across subdomains of the evidence map). Even so, compared with the additional more targeted search strings (EB+ search), the EW search (and the BR search) still appeared not to be sensitive enough (as suggested by “Congruence of evidence bases” section). It would appear that the risk for a lack of comprehensiveness [7] indeed is very real (and at the very same time also for too broad a scope for a systematic map).

### Opportunities for efficiency gains

In the face of expansive search results, any inefficiencies in the screening and coding process may get amplified to a point where a fair bit of efficiency gains (or very generous resources) may be needed for a mapping project to be tractable at all. OSM4 outlines a few tweaks that either helped me achieve substantial efficiency gains, or that I tried out to no avail but that I still deem potentially powerful. Taken together, the use of bibliographic APIs, data storage in a database management system rather than a spreadsheet, a parsimonious coding scheme, and a bespoke screening and coding tool (that is coupled to the database and offers efficient filtering and prepopulation of screening and coding fields) allowed efficiency gains for screening and coding of roughly one order of magnitude. Yet, despite these substantial efficiency gains, the mapping process that underpinned Egestabase was barely tractable. It would probably have been wise to explicitly deliberate whether it really was necessary to adhere to pertinent systematic mapping guidelines and standards, or if a more streamlined (yet still adequately rigorous) process might have been a better option.

### The importance of a good search strategy

Getting the search strategy right appears vital for a mapping endeavor to avoid ‘too little signal’ and ‘too much noise’. “Quantifying the effect of procedural differences” section suggested that the selection of search terms probably is the single most important factor in this regard—followed by search settings in terms of fields searched and Boolean operators applied to join search terms. This closely relates to search sensitivity and specificity, which implies a tradeoff between comprehensiveness of the map and viability of the mapping process. In other words, finding ways to maximize both sensitivity and specificity at the same time appears to be crucial (but not trivial)—and is what I will home in on here.

#### Combining broad searches with targeted searches

Evidence maps of broad research fields typically span several distinct subdomains within the research field to be mapped. The seemingly common practice of compiling relevant search terms into a single compound search string might thus be problematic in two ways. As suggested by “Search terms” section, compound search strings may for some subdomains find a larger fraction of potentially relevant studies in that subdomain than for other subdomains. “Constraining searches” section moreover suggests that search constraints intended to strike a better balance between sensitivity and specificity may not affect all subdomains equally. Taken together, this points to an issue that could be described as differential search sensitivity and specificity—and that so far appears to have been rather poorly theorized in the systematic mapping literature.

I reckon that disentangling broad compound search strings into a suite of narrower search strings has the potential to effectively tackle the issue of differential search sensitivity and specificity. On the one hand, it can be expected that search sensitivity becomes more balanced across subdomains (equally high proportions of the available relevant studies are found across all subdomains). On the other hand, the possibility to carefully fine-tune searches to individual subdomains (particularly in terms limitations to search fields, more restrictive search operators, and additional terms to further constrain the search) promises that the balance of search sensitivity and specificity is similar across subdomains.

Using narrow search strings directed at particular subdomains also implies that search hits can be used to prepopulate screening and coding fields. If a study is found through a very specific search, say on the alkaline dehydration of urine, said study should probably be included in the evidence base as a study that belongs to the category alkaline dehydration of urine. Thus, this approach to searching can contribute to a streamlined screening and coding process. After all, why would one first mix up all search terms—and consequentially search hits—just to then painstakingly disentangle them again? Wouldn’t it be better to keep things separate right from the start? It probably would.

On the flip side, handling many search strings is only realistic in combination with bibliographic APIs. More importantly, devising targeted search strings means that one first needs to have a good idea of what to target. With this said, broad compound search strings are still needed—though rather in terms of mapping out subdomains than mapping out the actual literature across subdomains. In other words, some sort of broad search string should still be part of the search strategy to ensure that categories that are not captured by the suite of more narrow search strings are not missed out on.

All in all, I would strongly recommend considering a combination of broad search strings (directed at establishing subdomains) and narrower search strings (directed at comprehensively finding evidence across individual subdomains)—which seems to be in line with the findings of Egan et al. [6] regarding the use of generic and specific search terms in systematic evidence synthesis. Further valuable guidance regarding the design of searches may be found in Bramer et al. [4].

#### Iterative approach to searching

The use of a broad compound search string to inform the delineation of a suite of more targeted search strings implies an iterative approach. In fact, trialing the search strategy—which is part of the scoping stage of systematic mapping [5, 12]—involves an iterative optimization of the search strategy. However, I would argue that feedback loops might need to extend beyond the scoping stage.

To begin with, a single compound search string is devised that is intended to capture subdomains as broadly as possible. While this search string should be characterized by high specificity, “Constraining searches” section suggests that (at least in the research domain discussed here) it is fine if sensitivity is low—as even the most restrictive search variant tested covered the overwhelming majority of subdomains despite missing out on the majority of relevant studies. Based on this limited set of search hits, one can then develop a first set of targeted search strings. After a fair bit of screening and coding (beyond the scoping stage), reviewers will get a sense of what subdomains one can expect ‘out there’—which in turn may surface the need for additional targeted search strings. Say one finds literature on the smolder combustion of feces. Domain knowledge suggests that this technology is also applicable to sewage sludge. If no associated literature is found, this may indicate that the suite of targeted search strings is not yet sufficiently comprehensive. At some point, no new subdomains will emerge, and all subdomains will be adequately covered by targeted search strings.

#### Grey literature

While it is generally recommended to include grey literature in evidence maps [7], the EW search (that forms the backbone of the EB evidence base) did not search for grey literature. This was motivated by experiences from the BR review—where reports written in English constituted a negligible fraction of the evidence found [15]. Moreover, the grey literature identified in the BR review seems to have consisted mainly of theses. Given the pressure for researchers to publish, it probably is fair to assume that evidence described in theses will eventually get published in one or several papers (or already has in the case of compilation theses). In the short term, some evidence might thus be missed by not searching relevant thesis repositories. At the same time, the inclusion of theses—notably compilation theses—may imply some level of double counting (unless multiple publications are combined into a single study where applicable). Above said, for knowledge domains where it is likely that very little is gained by searching grey literature, this extra effort should possibly not be a top priority.

#### Bibliographic databases

In systematic mapping, it is generally recommended to search multiple bibliographic databases [7]. As suggested by “Bibliographic databases” section, looking solely at what is indexed on different bibliographic databases, the choice of bibliographic database may for some knowledge domains have a rather moderate if not negligible effect on the mapping outcomes. But because of differences in search syntax across bibliographic databases, the choice of database may nevertheless have a larger effect on what is being retrieved through the searches than suggested by indexation. This issue can likely be mitigated (at least in part) by searching all available search fields.

For the evidence bases considered here, the gain of searching multiple bibliographic databases appears rather marginal compared to the more substantial gains through better search terms and settings. Going forward with Egestabase, I would thus be rather comfortable to focus on fine-tuning search strings and settings—for (in this specific case, but presumably also in other research fields in environmental sciences) it would appear that way more harm can be done by not getting search strings right than by not searching multiple bibliographic databases. As for which bibliographic database to choose, in the short run, I would search Elsevier Scopus (excellent API support and better coverage than Web of Science). In the long run, OpenAlex may become a good open access option (unless constrained by publisher copyright restrictions).

### The importance of quality checks

Working with multiple reviewers has the advantage that studies can be screened and coded by multiple reviewers and decisions compared. Ideally, this ensures high agreement across reviewers, so that screening and coding decisions are independent of the reviewer. This is why working individually is generally discouraged in systematic mapping [7]. While I fully back the theoretical considerations for having more than one reviewer, I think there are circumstances where the benefits of multiple reviewers may need to be weighed against the benefits of a single reviewer setting.

More specifically, using a bespoke screening and coding tool opens up a myriad of possibilities in terms of customized data processing, analysis, and visualization. On the flip side, there is a risk that only very few reviewers—perhaps even only one—can handle the tool in a productive way without substantial training in how to use the tool. In this very specific case, I think it may be worth considering a single reviewer configuration—provided a series of rigorous (post hoc) quality checks are implemented to hunt down potential inconsistencies and errors, which is what I will home in on here.

#### Checking the quality of screening and coding decisions

Even the best (team of) reviewers can make errors. Even the best machine learning algorithms are not perfect. Misclassifications can occur—for instance because a certain subdomain that could be classified in one way or another was not part of the set of studies classified by multiple reviewers, because a given study is hard to interpret, or simply out of negligence. This said, it appears to be almost inevitable to find some level of misclassification in any evidence base.

Especially when screening and coding are streamlined by filtering in combination with prepopulated screening and coding fields, it is likely that a few studies end up with a wrong classification. To spot these, excluded studies can for instance be screened again for combinations of terms that are common in included studies, and vice versa. Another way to hunt down this type of error could be by analyzing cross-citations. Are there any included (excluded) studies that are cited much less (more) frequently than most other included (excluded) studies? Are there any studies that are cited more frequently by a different subdomain than the one they were assigned to? In a similar vein, additional checks could involve information on authors, institutions, and source titles. There may also be subdomains with partly overlapping key terms—for instance the remobilization of phosphorus from return sludge for subsequent struvite precipitation versus the wet chemical extraction of phosphorus from sewage sludge for subsequent struvite precipitation. Such similar yet distinct subdomains can be expected to be prone to misclassification, and it might be indicated to subject them to an additional round of manual coding (ideally after consultation with relevant stakeholders and other domain experts). Above are just a few examples of possible plausibility and consistency checks that should be fairly simple to implement. Customized machine learning algorithms may also provide useful to facilitate plausibility and consistency checking—though probably harder to implement than those that can be performed directly in a database with a series of database operations.

#### Checking the quality of the search strategy

While issues with too low search specificity are easily spotted even without dedicated quality checks, detecting a lack of sensitivity is much trickier. The use of a benchmark list is a current best practice in systematic mapping for search development. It ideally involves consultation with domain experts to see if any subdomain is missing or underrepresented. However, for large mapping endeavors with thousands of potentially relevant papers and dozens of subdomains, such a benchmark list would need to be commensurately (perhaps even prohibitively) larger than for a small mapping project with only a few subdomains. In addition to a benchmark list, one could search for studies that are cited comparatively often by the included literature but were not found by the searches—this may indicate a problem with the sensitivity of the search.

### Better reporting of primary research

Researchers in the field of nutrient recovery and reuse from human excreta and domestic wastewater use many different words to refer to similar concepts (Table S1.3 in OSM1). Using more standardized terminology in the title, keyword and abstract fields can be expected to increase the likelihood that a given study is found during evidence synthesis and thus included in the resulting evidence base. In a similar vein, providing a clear description of what the study is about in the title and abstract would contribute to streamlining the evidence synthesis process—for it can be quite time consuming and frustrating when one must trawl through an entire paper to find clues on what the study is about.

### Systematic or not–the ultimate question?

Once more, it is not my intention to generally question systematic mapping or pertinent guidelines and standards. After all, systematic maps are meant to provide a transparent, robust and repeatable method to identify and collect relevant literature to a research question in policy or management—and who would not want their mapping to be transparent, robust and repeatable. I concur that most people would agree that a mapping process needs to be systematic in the sense of being done properly and avoiding systematic and other kinds of error and bias (as suggested by Hammersley [9]). My concerns are very specific.

On the one hand, I have experienced firsthand how handling large numbers of studies (tens of thousands of studies to be screened and thousands to be coded) can seriously bog down the mapping process. A good search strategy may help reduce the number of studies to be handled in the first place. In some cases, it may be necessary to reduce the scope of the map for the mapping process to be tractable. In other cases, reducing the scope of the map may not be desirable—thus requiring some degree of streamlining of the mapping process when a systematic mapping process turns out not to be tractable. This streamlining likely involves some tradeoffs in terms of what to prioritize. For this very specific case, I wonder whether there might be scope for some kind of guidance on the conduct and standards of ‘Rapid Mapping’ of evidence, similar to the available guidance on the conduct and standards of ‘Rapid Review’ of evidence (Sect. 10 of the CEE Guidelines and Standards for Evidence Synthesis in Environmental Management).

On the other hand, and this is potentially more serious, I suspect that it is much harder to assess how fit for purpose a search strategy is than how rigorous the rest of the mapping process is. Yet it appears to matter most for comprehensive and balanced mapping outcomes (at least in the research field considered here, but presumably also in other research fields in environmental sciences). To be more specific, it seems to be perfectly possible for a systematic map to be based on incomplete search strings that only partially align with eligibility criteria, and for the systematic map protocol and report to still pass peer-review. For instance, the BR systematic map mentions urine in its eligibility criteria and discusses the abundance of evidence on the recovery from urine versus other source streams. Interestingly, the term urine is not featured in the search string. As suggested by “Quantifying the effect of procedural differences” section, the impact of the search strategy on mapping outcomes (notably in terms of comprehensive and balanced coverage across subdomains) can be profound. Moreover, any shortcomings in the search strategy cannot be compensated for, no matter how rigorous the rest of the mapping process is. Admittedly, this is likely not an issue if the scope of the map is narrow and/or search terms are standardized in the research domain to be mapped. But when mapping large bodies of literature that lack standardized terminology, I reckon there is cause for concern.

### Outlook

Grounded in five evidence bases (from four previous reviews and one online evidence platform) on the recovery and reuse of nutrients in human excreta and domestic wastewater, the present paper attempted to estimate how a range of factors may have affected mapping outcomes. Based on this comparison, it articulated a number of reflections regarding what to prioritize and how to navigate tradeoffs in the face of too many studies and too little resources (and when it is deemed acceptable to deviate from systematic mapping guidelines and standards). This was done not from the perspective of an expert in evidence synthesis but from that of an expert in the knowledge domain being mapped. In this role, my concerns are twofold. First, I suppose that what I have referred to as ‘differential search sensitivity and specificity’ may jeopardize the rigor of mapping outcomes—especially so when rigorous post hoc quality checks are lacking. Second, I suspect that the quality of large maps crucially depends on the quality of the search strategy—yet this seems to be inherently harder to assess than the quality of the rest of the mapping process. Whether my reflections on these potential issues and how to tackle them bear any implications for adjusting systematic map guidelines and standards, the systematic evidence community is way more qualified to judge than I. In the meantime, I hope that the present paper can provide inspiration as to: (1) how to streamline the mapping process when a strict systematic mapping process appears not to be viable in the face of too much evidence and too little resources, (2) how to optimize search strategies to accomplish a more comprehensive and balanced map, and (3) what kind of rigorous (post hoc) quality checks may help minimize inconsistencies and errors. And of course, encourage domain experts to get more familiar with best practice in systematic mapping in case a systematic mapping process appears viable and desirable.

## Supplementary Information

Additional file 1: More Detailed Description of Evidence Bases

Additional file 2: Assessing the Robustness of Evidence Bases

Additional file 3: Details on Quantifying the Effect of Procedural Differences

Additional file 4: Opportunities for Efficiency Gains

## Data Availability

The evidence bases produced by the previous reviews are available in the respective publications. The Egestabase evidence base can be navigated online at www.egestabase.net.
