# Supplementary material for: Mapping large bodies of research in environmental sciences: insights from compiling evidence on the recovery and reuse of nutrients found in human excreta and domestic wastewater
Source: Environ Evid. 2025 Jul 14;14:13. doi: 10.1186/s13750-025-00366-5 (PMC12261714; doi:10.1186/s13750-025-00366-5)
Supplement: Supplementary file 1 — Additional file 1: More Detailed Description of Evidence Bases [file 13750_2025_366_MOESM1_ESM.pdf]

ENVIRONMENTAL EVIDENCE

Mapping Large Bodies of Research in Environmental Sciences: Insights from Compiling Evidence on the Recovery and Reuse of Nutrients Found in Human Excreta and Domestic Wastewater

Robin Harder

https://doi.org/10.1186/s13750-025-00366-5

SUPPORTING INFORMATION 1

Detailed Description of Evidence Bases

Table S1.1. Eligibility criteria applied in previous reviews.
N.B. Eligibility criteria for Egestabase are like for the EW review except that broad reviews were also included.

	Eligible Population(s)
BR	Municipal wastewater systems, including systems for treatment and disposal of wastewater fractions and wastewater-derived residues and products such as digestate, effluent water, etc. The wastewater fractions considered are blackwater (i.e. urine, faeces and flush water), greywater (i.e. wastewater from sinks, baths, washers etc.), mixed wastewater (i.e. blackwater and greywater mixed), urine, sludge reject water (i.e. liquid separated from anaerobically digested sludge) and other fractions such as faecal sludge. Both municipal and domestic onsite systems, as well as co-treatment of wastewater with other organic wastes were considered. No industrial or agricultural wastewater systems were considered. C and nutrient recycling from agricultural sources have been addressed in a related map.
SA	*NA*
UM	Source-separated urine was the primary population for our comprehensive mapping. Other wastewater fractions like brown water (e.g., faeces and flush water) or greywater (i.e., non-toilet plumbing systems, e.g., wastewater from sinks, baths, laundry, etc.) were excluded. Source-separated faeces/brown water, excreta/ blackwater, and greywater were excluded. Mixed wastewater (e.g., blackwater and greywater mixed, domestic and municipal) and sludge reject water from anaerobic digesters were also excluded. Papers dealing with mixed wastewater but also including source-separated urine were included, but only if they met the other inclusion criteria. The source of urine was limited to humans; therefore, studies dealing with urine from other sources, e.g., animals, were excluded. Urine could be real or synthetic, and it could also be fresh or hydrolyzed. The sources of urine included domestic on-site systems with urine diversion toilets and centralized and decentralized systems.
EW	Systems that manage human excreta or streams containing human excreta, notably domestic (municipal) wastewater. This includes systems that manage residues and products that are derived from human excreta or wastewater that contains human excreta, such as digestate, sewage sludge, treated effluent, etc. Synthetic wastewater intended to simulate the aforementioned streams was included. Both municipal and on-site systems were included, as well as co-treatment with other organic residuals. Systems that manage only greywater, stormwater, industrial wastewater (e.g., tannery wastewater), agricultural wastewater (e.g., milling wastewater) or animal manure were not included.

	Eligible Intervention(s)
BR	Any practice undertaken for the purposes of recovering and/or reusing carbon and/or nutrients in the wastewater management process including wastewater, sewage sludge, human urine etc., including the recovery of C in the form of energy. Ecotechnologies for recovery include the extraction or capture of the nutrients and/or C, through e.g. nutrient adsorption or bioenergy production. Reuse refers to a further use of the recovered nutrients and/or C, for example through the application of sludge or effluents to agricultural fields as fertilizers.
SA	*NA*
UM	The mapping focused on technologies for recovering plant nutrients from human urine and recycling these in the form of fertilizer (solid or liquid). Papers focusing on nutrient recovery were included in category 4. Other practices and processes that deal with human urine, but do not specifically recover and recycle nutrients in the form of fertilizer, were captured in the map by coding them into categories 1–3. Papers that did not meet the scope of the four cat- egories were excluded.
EW	Any technology or practice undertaken to facilitate the recirculation of plant nutrients, and possibly organic matter, to agriculture. Recirculation can take place either through direct reuse after treatment of human excreta or streams containing human excreta, or through products derived from the extraction of nutrients from human excreta or streams that contain or derive from human excreta. Practices that are undertaken for the sole purpose of recovering carbon (for instance as methane for energy purposes or as polyhydroxyalkanoate [PHA] for producing bioplastics) and water (for instance for potable reuse or industrial purposes) were excluded unless the practice encompasses simultaneous nutrient recovery or reuse (e.g., biochar production from sewage sludge, wastewater irrigation).

	Eligible Outcome(s)
BR	Described recovery or reuse of C and/or nutrients from the wastewater management processes. C outcomes included: energy, methane, biogas, soil C, soil organic C, total C, dissolved organic C, and organic matter, but also chemical oxygen demand and biological oxygen demand, which are proxies for C. Nutrient outcomes included: N compounds (N, nitrate, nitrite, ammonium) and P compounds (P, phosphate). However, reuse of recovered energy (such as using biogas for fuel) was not considered since the use of different forms of energy was outside of the scope of this map.
SA	*NA*
UM	The eligible outcomes of the technologies considered were nitrogen (N), phosphorus (P), and potassium (K) in the form of fertilizer. Therefore, the mapping focused solely on NPK recycling, as these nutrients are the main constituents of synthetic fertilizer, while technologies that only recover energy, carbon, salts, or other minerals and nutrients were not included. Note that the recovered nu- trients from urine might not be classified as a fertilizer by legislation and regulations in some jurisdictions, but within the scope of our mapping nutrients recovered by these technologies were counted as fertilizer, regardless of the legislative standpoint. The legislation and regulations context will be examined later in a follow-up TIS study.
EW	Products that contain plant nutrients originated from human excreta, with or without organic matter, and are suitable for reuse in agriculture, or as raw material to produce fertilizers.

	Eligible Study Type(s)
BR	Experimental or observational studies on N, P and/or C recovery and/or reuse. Literature reviews and commentary papers were excluded.
SA	*NA*
UM	Primary research publications, i.e., papers describing experimental and observational studies, were included. Book chapters describing experiments were also included. However, secondary research publications (e.g., literature, systematic and critical reviews, etc.) were excluded.
EW	Primary research that describes nutrient recovery technologies or the characteristics and reuse of recovered nutrients in agriculture. In addition to experimental studies at the lab, bench, pilot, or full scale, this also includes human health risk and sustainability assessments, as well as studies on user acceptance. In addition to primary research, we included reviews in cases they focus on specific pathways. Reviews with a general overview of reuse recovery were excluded.

Table S1.2: Search strings applied in previous reviews (continued on next page).

**SA**				
SA 1.1	A. Population (Source)<br>B. Intervention (Recirculation)		TITLE-ABS-KEY	(urine separation OR urine treatment)
SA 1.2	A. Population (Source)		TITLE-ABS-KEY	(domestic OR household OR municipal OR human)
		AND	TITLE-ABS-KEY	("black*water" OR "brown*water" OR "yellow*water")
SA 1.3	A. Population (Source)		TITLE-ABS-KEY	("ecological sanitation" OR "eco*san" OR "eco sanitation")
SA 1.4	A. Population (Source)		TITLE	(waste*water OR sanitation OR sludge)
	B. Intervention (Recirculation)	AND	TITLE	(recover* OR recycl*)
	C. Outcome (Target or Product)	AND	TITLE	(*nutrient OR nitrogen OR potassium OR phosphorus)
SA 1.5	A. Population (Source Modifier)		TITLE-ABS-KEY	(domestic OR household OR municipal OR human)
	A. Population (Source)	AND	TITLE	(sanitation OR waste*water)
	B. Intervention (Recirculation)	AND	TITLE	(farming OR agriculture* OR horticultur*)
SA 2.1	A. Population (Source)		TITLE	(sanitation OR urine OR yellow*water OR faeces OR "faecal matter" OR brown*water OR excreta OR black*water OR waste*water OR sewage OR sludge OR biosolid*)
	B. Intervention (Recirculation)	AND	TITLE	(recover* OR recycle* OR re*use OR valorisation OR farming OR agricultur* OR horticultur*)
	C. Outcome (Target or Product)	AND	TITLE	(*nutrient* OR phosph* OR nitrogen OR ammoni* OR potas* OR farming OR agricultur* OR horticultur*)
SA 2.2	A. Population (Source)		TITLE	(sanitation OR urine OR yellow*water OR faeces OR "faecal matter" OR brown*water OR excreta OR black*water OR waste*water OR sewage OR sludge OR biosolid*)
SA 2.2	B. Intervention (Recirculation)	AND	TITLE	(farming OR agricultur* OR horticultur*)
SA 2	EXCLUDES	AND NOT	TITLE	(animal OR livestock OR herbivore OR poultry OR swine OR pig* OR bovine OR cow* OR cattle OR dairy OR milk OR meat OR manure)
		AND NOT	TITLE	(hospital OR kidney OR blood OR child* OR women OR men OR metabolite)
		AND NOT	TITLE	(oil* OR petroleum OR coal OR mining OR mine OR paper* OR rubber OR pulp* OR *mill OR tannery OR beamhouse OR brewery OR dredg* OR concrete OR coking OR semiconductor OR electroplating OR pharmaceutic* OR salin* OR hydroxide OR *ethanol OR furnace OR silo* OR printing OR "disposable diaper*" OR manufactur* OR sugarbeet OR potato OR "dental metal")
		AND NOT	TITLE	(risk OR hazard OR prevalence OR disease OR health OR occupation* OR work* OR toxic* OR schistosomiasis OR virologic* OR bacteriologic* OR hygien* OR epidemiolog* OR infection OR exposure))
		AND NOT	TITLE	(reservoir OR hydrogeologic OR kinetic* OR speciation OR uptake OR dissipation OR morphology OR fate OR mobility OR monitoring OR runoff OR bloom*)
		AND NOT	TITLE	(impact* OR planning OR evaluation OR determination OR assess* OR model* OR effect OR policy OR consequence* OR attitude OR perce* OR preference)
		AND NOT	TITLE	(indust*) AND NOT (municipal OR commun* OR domestic OR city OR urban OR campus OR region*)
		AND NOT	TITLE	(pollutant* OR contamina* OR pah OR las OR "linear alkylbenzene sulfonate" OR phenol* OR pae OR pcb OR dioxin OR furan OR polybrom* OR *fluor* OR *samine OR pathogen* OR "heavy metal*" OR virus OR bacteri* OR helminth* OR giardi* OR salmonella OR estrogen OR pcdd OR antibiotic* OR pesticide* OR aromatic OR hydrocarbon* OR triclo* OR surfactant* OR ibuprofen OR estrone OR estradiol OR phthalate OR drug* OR glyphosate) AND NOT (remov* OR inactivat* OR destr*)
		AND NOT	TITLE	("*cult* waste*" OR "*farming waste*" OR "shrimp waste*" OR "fish waste*" OR "household garbage" OR "phosph* sludge" OR "phosphoric acid sludge" OR "alum* sludge")
		AND NOT	TITLE	(social OR economic) AND NOT (environmental)

**BR**				
BR	A. Population (Source)		TITLE-ABS-KEY	(wastewater OR "waste water" OR "storm water" OR stormwater OR blackwater OR "black water" OR greywater OR "grey water" OR graywater OR "gray water" OR sludge OR septage OR sewage OR "organic waste*" OR "septic sludge" OR sewerage* OR digestate* OR "toilet waste")
	B. Intervention (Recirculation)	AND	TITLE-ABS-KEY	(return OR recovery OR recover OR conversion OR convert OR circulate OR circular OR reuse OR recycle)
	C. Outcome (Target or Product)	AND	TITLE-ABS-KEY	("organic carbon" OR DOC OR "organic C" OR "organic matter" OR nutrient* OR nitrogen OR nitrate OR nitrite OR ammoni* OR phosphorus OR phosphate)

Table S1.2: Search strings applied in previous reviews (continued from previous page).

**UM**				
UM 01	A. Population (Source)		TITLE-ABS-KEY	(urine)
	B. Intervention (Recirculation)	AND	ALL-FIELDS	(recover*)
	C. Outcome (Target or Product)	AND	ALL-FIELDS	(nutrient*)
UM 02	A. Population (Source)		TITLE-ABS-KEY	(urine OR yellowwater OR “yellow water”)
	B. Intervention (Recirculation)	AND	TITLE-ABS-KEY	(recover* OR circul* OR recycl*)
	C. Outcome (Target or Product)	AND	TITLE-ABS-KEY	(nutrient* OR nitrogen OR urea OR ammonia OR ammonium OR phosphorus OR phosphate OR potassium OR fertili* OR struvite)
UM 03	A. Population (Source)		TITLE-ABS-KEY	(urine OR urinal OR yellowwater OR yellow water OR yellow-water)
	B. Intervention (Recirculation)	AND	TITLE-ABS-KEY	(recover* OR *circul* OR reus* OR recycl* OR fertili* OR fertigat* OR conditioner* OR amendment* OR agricultur* OR land application*)

**EW**				
EW	A. Population (Source)		TITLE-ABS-KEY	(WASH OR sanitation OR watsan OR ecosan OR toilet* OR latrine* OR urinal* OR urine OR feces OR faeces OR excreta OR excrement* OR “human waste” OR “human manure” OR humanure OR “night soil” OR night-soil OR yellowwater OR “yellow water” OR brownwater OR “brown water” OR blackwater OR “black water” OR septage OR sewage OR sewerage OR wastewater OR “waste water” OR digestate* OR effluent* OR sludge OR biosolid*)
	B. Intervention (Recirculation)	AND	TITLE-ABS-KEY	(recover* OR *circul* OR reus* OR recycl* OR fertili* OR fertigat* OR conditioner* OR amendment* OR agricultur* OR “land application*”)
	C. Outcome (Target or Product)	AND	TITLE-ABS-KEY	(organic* OR nutrient* OR biosolid OR nitrogen OR urea OR ammonia OR ammonium OR phosphorus OR phosphate OR phosphoric OR potassium OR potash OR fertili* OR *char OR *compost OR ash* OR biomass OR struvite OR vivianite OR worm*)))

Table 1.3. List of search terms used across search strategies. Similar search term 'variations' can be grouped into associated 'basic' search terms that are then associated with concepts. N.B. Not all concepts, search terms and variations are used in all search strings of previous reviews.

	CONCEPT	BASIC SEARCH TERM	SEARCH TERM VARIATIONS
POPULATION	sanitation	sanitation	sanitation, ECOSAN, WATSAN, WASH
		toilet	toilet, toilets
		latrine	latrine, latrines
		urinal	urinal, urinals
		privy	privy, privies
	human urine	urine	urine
		yellowwater	yellowwater, yellow water, yellow-water
	human feces	feces	feces, faeces, feaces
		brownwater	brownwater, brown water, brown-water
	human excreta	fecal matter	fecal matter, faecal matter, feacal matter
		excreta	excreta
		excrement	excrement, excrements
		night soil	night soil, nightsoil, night-soil
		human manure	human manure, human manures, humanure
		human waste	human waste, human wastes
		toilet waste	toilet waste, toilet wastes
		blackwater	blackwater, black water, black-water
	wastewater	septage	septage, septic tank, septic sludge, fecal sludge
		sewerage	sewerage
		sewage	sewage
		wastewater	wastewater, waste water, waste-water
		slurry	slurry, slurries
		sludge	sludge, sludges
		biosolid	biosolid, biosolids
		digestate	digestate, digestates
		effluent	effluent, effluents
INTERVENTION	treat	convert	convert, converted, converting, conversion
		treat	treat, treating, treated, treatment
		hygienize	hygienize, disinfect, sanitize
		disinfect	disinfect, disinfected, disinfecting, disinfection
		sanitize	sanitize, sanitized, sanitizing, sanitization
		pasteurize	pasteurize, pasteurized, pasteurizing, pasteurization
		degrade	degrade, degraded, degrading, degradation
		remove	remove, removed, removing, removal
		inactivate	inactivate, inactivated, inactivating, inactivation
		storage	store, stored, storing, storage
		oxidation	oxidize, oxidized, oxidizing, oxidation, microwave, microwaving, irradiation
		water extraction	concentration, water extraction, distillation, evaporation, dehydration, drying, freezing and thawing
		crystallization	precipitation, crystallization, electrocoagulation
		sorption	sorption, adsorption, desorption, sorbent, ion exchange, ion exchanger
		membrane separation	filtration, membrane, microfiltration, ultrafiltration, nanofiltration, reverse osmosis, forward osmosis
		biomass growth	photoreactor, microbial, algal, algae, bacterial, bacteriae, spirulina, chlorella, plankton
		electrochemical system	electrochemical, electrokinetic, electrolysis, fuel cell, diffusion cell, deonization
		anaerobic decomposition	digest, digested, digesting, digestion, fermentation
		aerobic decomposition	compost, composting, vermicompost, vermicomposting, black soldier fly, fly larvae, worm, insect
		thermal decomposition	thermal, incineration, smouldering, combustion, pyrolysis, carbonisation, liquefaction, gasification
		hydrothermal decomposition	supercritical water, hydrothermal
	recover	recover	recover, recovered, recovering, recovery
		reclaim	reclaim, reclaimed, reclaiming, reclamation
		extract	extract, extracted, extracting, extraction
		capture	capture, captured, capturing
		strip	strip, stripped, stripping
		volatilize	volatilize, volatilized, volatilizing, volatilization
	recycle	recycle	recycle, recycled, recycling
		circulate	circular, circulate, circulated, circulating, circulation
		recirculate	recirculate, recirculated, recirculating, recirculation
	reuse	reuse	reuse, reused, reusing
		use	use, used, using, usage
		utilize	utilize, utilized, utilizing, utilization, utilise, utilised, utilising, utilisation
		valorize	valorize, valorized, valorizing, valorization, valorise, valorised, valorising, valorisation
		apply	apply, applied, applying, application
		amend	amend, amended, amending, amendment
		fertilize	fertilize, fertilized, fertilizing, fertilization
		fertigate	fertigate, fertigated, fertigating, fertigation
		irrigate	irrigate, irrigated, irrigation
		return	return, returned, returning
	agriculture	agriculture	agriculture, agricultural
		horticulture	horticulture, horticultural
		farming	farming, farm, farms
		agronomic	agronomic, agronomy
		land	land
		soil	soil, soils
		crop	crop crops
OUTCOME	nutrient	nutrient	nutrient, nutrients, macronutrient, macronutrients, micronutrient, micronutrients
		nitrogen	nitrogen, urea, ammonia, ammonium, nitrate, nitrite
		phosphorus	phosphorus, phosphorous, phosphoric
		potassium	potassium, potash
	product	crop fertilizer	fertiliser, fertilizer, amendment, conditioner
		precipitates	phosphate, struvite, hydroxyapatite, vivianite
		organic matter	biomass, digestate, sewage sludge, biosolid, char, compost
		protein biomass	microalgae, worm, worms
		ash	ash, ashes

Table 1.4. Coverage of concepts and search terms in previous reviews.
N.B. Previous reviews may differ regarding search term alternatives used.

	CONCEPT	BASIC SEARCH TERM	BR	SA	UM	EW	EB+
POPULATION	sanitation	sanitation		x		x	x
		toilet				x	x
		latrine				x	x
		urinal				x	x
		privy					x
	human urine	urine		x	x	x	x
		yellowwater		x	x	x	x
	human feces	feces		x		x	x
		brownwater		x		x	x
	human excreta	fecal matter		x			
		excreta		x		x	x
		excrement					x
		night soil				x	x
		human manure				x	x
		human waste				x	x
		toilet waste	x			(x)	(x)
		blackwater	x	x		x	
	wastewater	septage	x			x	x
		sewerage	x			x	x
		sewage	x	x		x	x
		wastewater	x	x		x	x
		slurry					x
		sludge	x	x		x	x
		biosolid		x		x	x
		digestate	x			x	x
		effluent				x	x
INTERVENTION	treat	convert	x				
		treat					x
		hygienize					x
		disinfect					x
		sanitize					x
		pasteurize					x
		degrade					x
		remove					x
		inactivate					x
		storage					x
		oxidation					x
		water extraction					x
		crystallization					x
		sorption					x
		membrane separation					x
		biomass growth					x
		electrochemical system					x
		anaerobic decomposition					x
		aerobic decomposition					x
		thermal decomposition					x
		hydrothermal decomposition					x
	recover	recover	x	x	x	x	x
		reclaim					x
		extract					x
		capture					x
		strip					x
		volatilize					x
	recycle	recycle	x	x	x	x	x
		circulate	x		x	x	x
		recirculate				(x)	x
	reuse	reuse	x	x		x	x
		use					x
		utilise					x
		valorise		x			x
		apply				x	x
		amend				x	x
		fertilise				x	x
		fertigate				x	x
		irrigate					x
		return	x				
	agriculture	agriculture		x		x	x
		horticulture		x			x
		farming		x			
		agronomic					x
		land					x
		soil					x
OUTCOME	nutrient	nutrient	x	x	x	x	x
		nitrogen	x	x	x	x	x
		phosphorus	x	x	x	x	x
		potassium		x	x	x	x
	product	fertiliser			x	x	x
		precipitates			x	x	x
		organic matter	x			x	x
		protein biomass				x	x
		ash				x	x

Table 1.5. Coding schemes and elements for previous reviews and Egestabase.

(a) Elements distinguished in the **topic** dimension.

**BR**	
Recovery	Reuse

**SA**	
Recovery	

**UM**	
Technologies for Recovery of Nutrients from Urine<br>Urine Use in Soil and Agricultural Applications	Pharmaceutical and Pathogen Removal from Urine<br>Source Separation and Urine Diversion

**EW**	
Technology<br>Reuse	Product<br>User Acceptance

**EB**	
Technology<br>Reuse	Product<br>User Acceptance

(b) Elements distinguished in the **source stream** dimension.

**BR**	
Urine<br>Blackwater<br>Wastewater (mixed/conventional)<br>Sludge liquid phase	Sludge<br>Other<br>Greywater

**SA**	
Urine<br>Feces<br>Brownwater<br>Excreta<br>Blackwater	Fecal Sludge<br>Wastewater<br>Sewage Liquid Fraction<br>Sewage Sludge<br>Sewage Sludge Ash

**UM**	
Urine	

**EW**	
Human Urine<br>Yellowwater<br>Human Feces<br>Brownwater<br>Human Excreta<br>Blackwater	Fecal Sludge<br>Septage<br>Nappy Waste<br>Sewage Effluent or Side Stream<br>Sewage Sludge<br>Sewage Sludge Ash

**EB**	
Human Urine<br>Yellowwater<br>Human Feces<br>Brownwater<br>Human Excreta	Blackwater<br>Fecal Sludge or Septage<br>Sewage Effluent or Side Stream<br>Sewage Sludge<br>Sewage Sludge Ash

(c) Elements distinguished in the **technology** dimension.

**BR**	
Macroalgae cultivation<br>Microalgae cultivation<br>Biomass production directly in substrate, other than algae<br>Struvite precipitation<br>P precipitation, other than struvite<br>Membrane filtration<br>Electrodialysis<br>Adsorption<br>Ammonia stripping<br>Leaching<br>Thermal ash recovery<br>Anaerobic bioreactor<br>Anaerobic digestion	Composting<br>Vermicomposting<br>Incineration<br>Pyrolysis<br>Torrefaction<br>Other thermal treatment<br>Hydrothermal carbonization<br>Hydrothermal liquefaction<br>Supercritical water gasification<br>Microbial fuel cell<br>Microbial electrolysis cell or similar<br>Other

(continued on next page)

(continued from previous page)

SA	
STAB :: Acidification<br>STAB :: Alkalinisation<br>STAB :: Chemical Oxidation<br>STAB :: Electrochemical Potential<br>STAB :: Lactic Acid Fermentation<br>STAB :: Nitrification<br>PA.RED :: Ammonia Sanitisation<br>PA.RED :: Advanced Oxidation Processes<br>PA.RED :: Desiccation<br>PA.RED :: Lactic Acid Fermentation<br>PA.RED :: Lime Treatment<br>PA.RED :: Pasteurisation<br>PA.RED :: Storage<br>PA.RED :: Thermal Storage<br>OP.RED :: Biological Treatment<br>OP.RED :: Advanced Oxidation<br>OP.RED :: Electrodialysis<br>OP.RED :: Nanofiltration<br>HM.RED :: Heavy Metal Removal<br>ASH.TR :: Smelt Gasification<br>ASH.TR :: Thermo-Chemical Treatment<br>ASH.TR :: Thermo-Reductive Treatment<br>V.RED :: Evaporation<br>V.RED :: Distillation<br>V.RED :: Freeze Concentration<br>V.RED :: Electrodialysis<br>V.RED :: Forward Osmosis	V.RED :: Reverse Osmosis<br>V.RED :: Membrane Distillation<br>BMGR :: Photobioreactor (Microalgae)<br>BMGR :: Photobioreactor (Algae)<br>BMGR :: Photobioreactor (Zooplankton)<br>BMGR :: Photobioreactor (Cynobacteria)<br>BMGR :: Macrophyte Treatment (Duckweed)<br>CRST :: Controlled Precipitation<br>CRST :: Precipitation in MFC<br>CRST :: Granulation in UASB<br>SORP :: Organic Sorbent Material<br>SORP :: Inorganic Sorbent Material<br>SORP :: Synthetic Sorbent Material<br>STRI :: Air Stripping<br>STRI :: Air Stripping (Electrochemical Cell)<br>STRI :: Air Stripping (Microbial Electrolysis Cell)<br>STRI :: Air Stripping (Microbial Fuel Cell)<br>STRI :: Distillation<br>P.EXT :: Remobilization in Return Sludge<br>BDEC :: Lactic Acid Fermentation<br>BDEC :: Composting<br>BDEC :: Vermicomposting<br>TDEC :: Incineration<br>TDEC :: Smoulder Combustion<br>TDEC :: Pyrolysis<br>TDEC :: Hydrothermal Liquefaction

UM	
Urine stabilization techniques<br>Storage<br>Freezing and thawing<br>Evaporation<br>Alkaline dehydration<br>Nitrification/distillation<br>Membrane<br>Forward/reverse osmosis<br>Forward osmosis & Membrane distillation	Microalgae biotechnology<br>Struvite precipitation/crystallization<br>Struvite precipitation & Adsorption<br>Struvite precipitation & Ammonia stripping<br>Sorption: Ion exchange, absorption, adsorption<br>Ammonia/air stripping<br>Ammonia stripping & Adsorption<br>Microbial electrochemical technologies

EW	
TRT_STA : Urea Hydrolysis<br>TRT_STA : Acidification<br>TRT_STA : Alkalinisation<br>TRT_STA : Nitrification<br>TRT_STA : Stabilisation – Not Further Specified<br>TRT_CON : Advanced Oxidation<br>TRT_CON : Ammonia Sanitisation<br>TRT_CON : Contaminant Sorption<br>TRT_CON : Drying<br>TRT_CON : Electrodialytic Treatment<br>TRT_CON : Lactic Acid Fermentation<br>TRT_CON : Membrane Filtration<br>TRT_CON : Metal Leaching<br>TRT_CON : Pasteurisation<br>TRT_CON : Storage<br>TRT_CON : Thermal Hydrolysis<br>TRT_CON : Thermal Treatment<br>TRT_CON : Thermochemical Treatment<br>TRT_CON : Contaminant Reduction – Not Further Specified<br>WEX_VAP : Alkaline Dehydration<br>WEX_VAP : Crystallisation Evaporation<br>WEX_VAP : Distillation<br>WEX_VAP : Evaporation<br>WEX_VAP : Membrane Distillation (Water Extraction)<br>WEX_VAP : Nitrification-Distillation<br>WEX_VAP : Passive Evaporation<br>WEX_VAP : Solar Thermal Distillation<br>WEX_VAP : Solar Thermal Evaporation<br>WEX_VAP : Vacuum Evaporation<br>WEX_VAP : Vertical Gauze Sheet Evaporation	NUT_PRE : Electrocoagulation<br>NUT_PRE : Granulation<br>NUT_PRE : N Crystallisation<br>NUT_MEM : Bio/Electrochemical System (Nutrient Extraction)<br>NUT_MEM : Electrodialysis (Nutrient Extraction)<br>NUT_MEM : Forward Osmosis (Nutrient Extraction)<br>NUT_MEM : Microfiltration (Nutrient Extraction)<br>NUT_MEM : Nanofiltration (Nutrient Extraction)<br>NUT_MEM : Polymer Inclusion Membrane (Nutrient Extraction)<br>NUT_MEM : Reverse Electrodialysis (Nutrient Extraction)<br>NUT_MEM : Reverse Osmosis (Nutrient Extraction)<br>NUT_MEM : Ultrafiltration (Nutrient Extraction)<br>NUT_SOR : Sorption to Sorbent<br>NUT_SOR : Sorption with Regeneration<br>NUT_AMM : Air Stripping<br>NUT_AMM : Membrane Stripping<br>NUT_AMM : Steam Distillation<br>NUT_AMM : Struvite Calcination<br>NUT_AMM : Thermal Drying<br>NUT_AMM : Vacuum Stripping<br>NUT_PEX : Phosphorus Extraction – Elution to Solution<br>NUT_PEX : Phosphorus Extraction – Solvent Extraction<br>NUT_PEX : Phosphorus Extraction – Vaporisation<br>NUT_PEX : Phosphorus Extraction – with Precipitation<br>BDC_DIG : Aerobic Digestion<br>BDC_DIG : Anaerobic Digestion<br>BDC_DIG : Fermentation<br>BDC_CMP : Bacterial Composting<br>BDC_CMP : Fungal Composting<br>BDC_CMP : Insect Composting

(continued on next page)

(continued from previous page)

WEX_MEM : Bio/Electrochemical System (Water Extraction)<br>WEX_MEM : Electrodialysis (Water Extraction)<br>WEX_MEM : Forward Osmosis (Water Extraction)<br>WEX_MEM : Membrane Distillation (Water Extraction)<br>WEX_MEM : Nanofiltration (Water Extraction)<br>WEX_MEM : Reverse Osmosis (Water Extraction)<br>WEX_MEM : Ultrafiltration (Water Extraction)<br>WEX_FRT : Freeze Concentration<br>WEX_PHY : Dehydration<br>WEX_PHY : Dewatering<br>WEX_PHY : Drying<br>WEX_PHY : Vortex Separation<br>NUT_PRO : Protein Growth – Aquatic Macrophytes<br>NUT_PRO : Protein Growth – Aquatic Microphytes<br>NUT_PRO : Protein Growth – Terrestrial Organisms<br>NUT_PRE : Biomineralisation<br>NUT_PRE : Chemical Precipitation<br>NUT_PRE : Coagulation<br>NUT_PRE : Electrochemical Precipitation	BDC_CMP : Terra Preta<br>BDC_CMP : Worm Composting<br>TDC_THE : Gasification<br>TDC_THE : Incineration<br>TDC_THE : Pyrolysis<br>TDC_THE : Smouldering<br>TDC_THE : Torrefaction<br>TDC_HYD : Hydrothermal Carbonisation<br>TDC_HYD : Hydrothermal Gasification<br>TDC_HYD : Hydrothermal Humification<br>TDC_HYD : Hydrothermal Liquefaction<br>TDC_HYD : Hydrothermal Mineralisation<br>TDC_HYD : Hydrothermal Oxidation<br>TDC_HYD : Hydrothermal Treatment<br>TDC_HYD : Wet Oxidation<br>TDC_HYD : Subcritical Water<br>TDC_HYD : Subcritical Water Gasification<br>TDC_HYD : Supercritical Water Gasification<br>TDC_HYD : Supercritical Water Oxidation

EB	
PTR_TRT Biological Treatment<br>PTR_TRT Dewatering<br>PTR_TRT Sludge Treatment<br>PTR_STA Urea Hydrolysis<br>PTR_STA Urea Stabilization<br>PTR_STA Ammonia Stabilization<br>PTR_STA Ammonia Oxidation<br>CON_REM Contaminant Sorption<br>CON_REM Membrane Filtration<br>CON_REM Thermochemical Ash Treatment<br>CON_DEC Ammonia Sanitisation<br>CON_DEC Oxidation<br>CON_DEC pH Modification<br>CON_DEC Storage<br>CON_DEC Thermal Treatment<br>WEX_VAP Evaporation<br>WEX_VAP Distillation<br>WEX_MEM Hydraulic Gradient<br>WEX_MEM Osmotic Gradient<br>WEX_MEM Thermal Gradient<br>WEX_FRT Freeze Concentration<br>NUT_PRO Macrophyte<br>NUT_PRO Microphyte<br>NUT_PRE Biomineralisation<br>NUT_PRE Chemical Precipitation<br>NUT_PRE Electrochemical Precipitation<br>NUT_PRE Granulation	NUT_MEM Hydraulic Gradient<br>NUT_MEM Osmotic Gradient<br>NUT_MEM Electrochemical Gradient<br>NUT_SOR Adsorption to Sorbent<br>NUT_SOR Adsorption and Desorption<br>NUT_AMM Air Stripping<br>NUT_AMM Membrane Stripping<br>NUT_AMM Steam Stripping<br>NUT_AMM Vacuum Stripping<br>NUT_AMM Thermal Processes<br>NUT_PEX Biosolubilisation<br>NUT_PEX Wet Chemical Extraction<br>NUT_PEX Electrochemical Extraction<br>NUT_PEX Thermal Extraction<br>BDC_DIG Fermentation<br>BDC_DIG Anaerobic Digestion<br>BDC_CMP Composting<br>BDC_CMP Insect Composting<br>BDC_CMP Worm Composting<br>BDC_CMP Terra Preta<br>TDC_THE Combustion<br>TDC_THE Pyrolysis<br>TDC_THE Gasification<br>TDC_HYD Hydrothermal Carbonisation<br>TDC_HYD Hydrothermal Gasification<br>TDC_HYD Hydrothermal Liquefaction<br>TDC_HYD Hydrothermal Oxidation

(d) Elements distinguished in the **target** dimension.

BR	
N<br>P	C<br>Energy

SA	
Nutrients<br>Urea-N<br>NH4-N	P<br>K

UM	

EW	
Nutrients<br>N	P<br>K

EB	
Nutrients<br>N	P<br>K

(continued on next page)

(continued from previous page)

(e) Elements distinguished in the **product** dimension.

**BR**	
Effluents<br>Biosolids/sludge/compost	Biochar, biocoal or adsorption medium<br>Other products

**SA**	
Ammonium Chloride<br>Ammonium Nitrate<br>Ammonium Sulfate<br>Ammonium Water<br>Ash<br>Biochar<br>Biomass<br>Compost<br>Dehydrated Faeces<br>Hygienised Blackwater	Hygienised Effluent<br>Hygienised Faecal Sludge<br>Hygienised Sludge<br>Precipitate<br>Slag<br>Solution<br>Sorbent<br>Terra Preta<br>Vermicompost

**UM**	

**EW**	
LIQ_NUT : Urine – Treated | Concentrated<br>LIQ_NUT : Effluent – Treated | Concentrated<br>LIQ_NPK : Nutrient Solution – N<br>LIQ_NPK : Nutrient Solution – P<br>LIQ_NPK : Nutrient Solution – N | P | K<br>INO_PRE : Ammonium Crystals<br>INO_PRE : Methylene Urea Crystals<br>INO_PRE : Calcium Phosphate<br>INO_PRE : Struvite<br>INO_PRE : K-Struvite<br>INO_PRE : Iron Phosphate<br>INO_PRE : Other Phosphates<br>INO_ASH : Ash	ORG_FDM : Biochar<br>ORG_FDM : Bioslurry<br>ORG_FDM : Biosolid<br>ORG_PRO : Microalgae<br>ORG_PRO : Macrophytes<br>ORG_PRO : Fish<br>ORG_PRO : Plant<br>OTH_SOR : Nutrient-Enriched Sorbent – Organic<br>OTH_SOR : Nutrient-Enriched Sorbent – Mineral<br>OTH_SOR : Nutrient-Enriched Sorbent – Synthetic<br>OTH_FER : Organomineral Fertilizer<br>OTH_FER : Biostimulant

**EB**	
NRS : urine<br>NRS : effluent<br>NRS : bioliquid<br>NRS : nitrogen concentrate<br>PRE : precipitate<br>ASH : ash	FDM : bioslurry<br>FDM : biosolid<br>FDM : biochar<br>PRB : protein<br>SOR : sorbent (nutrient-enriched)

(f) Elements distinguished in the **reuse** dimension.

**BR**	
Irrigation	Reuse

**SA**	

**UM**	

**EW**	
Plant Fertilizer<br>Soil Conditioner	Feed Protein

**EB**	
Crop Fertilizer<br>Fertilizer Production	Animal Feed<br>Microbial Protein Production
