# Supplementary material for: Mapping large bodies of research in environmental sciences: insights from compiling evidence on the recovery and reuse of nutrients found in human excreta and domestic wastewater
Source: Environ Evid. 2025 Jul 14;14:13. doi: 10.1186/s13750-025-00366-5 (PMC12261714; doi:10.1186/s13750-025-00366-5)
Supplement: Supplementary file 2 — Additional file 2: Assessing the Robustness of Evidence Bases [file 13750_2025_366_MOESM2_ESM.pdf]

ENVIRONMENTAL EVIDENCE

Mapping Large Bodies of Research in Environmental Sciences: Insights from Compiling Evidence on the Recovery and Reuse of Nutrients Found in Human Excreta and Domestic Wastewater

Robin Harder

https://doi.org/10.1186/s13750-025-00366-5

SUPPORTING INFORMATION 2

Assessing the Robustness of Evidence Bases

Assessing the robustness of the evidence bases in terms of screening and coding decisions requires a pairwise comparison at the level of individual studies. To this end, it seems expedient to compare screening and coding outcomes of the BR, SA, UM, and EW reviews with those underpinning the EB online evidence platform – as all studies considered for inclusion in those reviews (except for the grey literature considered in the BR review) were also considered for inclusion in Egestabase. Overall agreement based on this comparison is summarized in Table S2.1.

Table S2.1 Agreement of screening and coding outcomes across evidence bases. * Technology category only. INCL = Included in respective evidence base. EXCL = Excluded in respective evidence base.

(a)			(b)			(c)			(d)		
BR			SA			UM			EW		
EXCL	INCL	INCL	EXCL	INCL	INCL	EXCL	INCL	INCL	EXCL	INCL	INCL
(63)<br>134<br>6	2<br>234<br>14	2<br>93<br>2	NA<br>NA<br>NA	12<br>248<br>22	0<br>15<br>3	(43)<br>54<br>1	24<br>289 *<br>2	15<br>188<br>1	(705)<br>705<br>0	210<br>10 175<br>6	(745)<br>745<br>0
INCL	INCL	EXCL	INCL	INCL	EXCL	INCL	INCL	EXCL	INCL	INCL	EXCL
EB			EB			EB			EB		

(a) BR versus EB. A total of 93 studies were included in the BR but not in the EB evidence base. Based on the respective eligibility criteria, only 4 of these studies appear to be potentially wrong screening outcomes (2 in the BR and 2 in the EB evidence base). This suggests that the studies included in the BR but not in the EB evidence base essentially are those that are about the recovery of carbon (including energy) without concurrent recovery of nutrients – these studies were eligible in the BR review but not for EB. Moreover, there are 134 studies included in the EB but not in the BR evidence base. It would appear that 63 of these studies (of which 70% are on irrigation with effluents or reuse of biosolids) could possibly also have been included in the BR evidence base while 6 should have been excluded from the EB evidence base. Coding of includes appears to be reliable in both evidence bases.

(b) SA versus EB. A total of 15 studies were included in the SA but not in the EB evidence base. Of these studies, 3 could in fact have been included in the EB evidence base. Studies that were excluded from the SA but included in the EB evidence base were impossible to identify as neither search hits nor excludes were tracked in the SA review. Coding of includes appears to be sufficiently reliable in both evidence bases.

(c) UM versus EB. A total of 188 studies were included in the UM but not in the EB evidence base. Based on the respective eligibility criteria, only 16 of these studies appear to be potentially wrong screening outcomes (15 in the UM and 1 in the EB evidence base). This suggests that the studies included in the UM but not in the EB evidence base essentially are those that are broadly about source separation and urine diversion – these studies were eligible in the UM review but not for EB. At the same time, it would appear that 22 studies could have been excluded from the UM evidence base (as they seem to not be about human urine). Moreover, there are 54 studies that were excluded from the UM but included in the EB evidence base. It would appear that 43 studies may have been included in the UM and 1 should have been excluded from the EB evidence base. Coding of includes appears to be sufficiently reliable in both evidence bases.

(d) EW versus EB. A total of 745 studies were included in the EW but not in the EB evidence base. These mostly are studies that are on technologies used for nutrient recovery but do not describe the fate of nutrients during treatment or the reuse of the nutrient rich product after treatment – the EW review was rather inclusive in this regard and thus seems to have included studies that may as well have been excluded. Conversely, 705 studies were excluded from the EW review but included in the EB evidence base. It is likely that these studies could possibly have been included also in the EW evidence base. Coding of includes appears to be reliable in both evidence bases.

In the BR evidence base, included studies appear to reliably be studies that ought to be included, while some of the excluded studies could possibly have been included. In the SA evidence base, included studies appear to also reliably be studies that ought to be included, while many of the studies that were not screened would have been included if screened – but were not needed as the scope of the SA review was to find at least one study per coding category rather than as many as possible. In the UM evidence base, some of the includes could possibly have been excluded while some of the excludes could possibly have been included. Also, there seems to be a few glitches in coding – none of which are likely to jeopardize the quality and validity of the assessment of knowledge evolution that was the main goal of the UM review. In the EW evidence base, there seems to be some irregularities both in terms of including studies that may as well have been excluded, and in terms of studies that were excluded but may as well have been included – but still relatively few given that over 130 000 studies were screened. Also, there seem to be a few glitches in coding – mainly related to coding categories that are hard to tell apart. These minor inconsistencies seem to have been corrected for in the EB evidence base (the compiling of which involved a partial validation of the EW coding).

Taken together, screening and coding irregularities that emerged upon pairwise comparison of evidence bases at the level of individual studies, expressed as fraction of studies included in the respective evidence base, are estimated to be below 5% for all evidence bases.
