# Supplementary material for: Mapping large bodies of research in environmental sciences: insights from compiling evidence on the recovery and reuse of nutrients found in human excreta and domestic wastewater
Source: Environ Evid. 2025 Jul 14;14:13. doi: 10.1186/s13750-025-00366-5 (PMC12261714; doi:10.1186/s13750-025-00366-5)
Supplement: Supplementary file 3 — Additional file 3: Details on Quantifying the Effect of Procedural Differences [file 13750_2025_366_MOESM3_ESM.pdf]

ENVIRONMENTAL EVIDENCE

Mapping Large Bodies of Research in Environmental Sciences: Insights from Compiling Evidence on the Recovery and Reuse of Nutrients Found in Human Excreta and Domestic Wastewater

Robin Harder

https://doi.org/10.1186/s13750-025-00366-5

SUPPORTING INFORMATION 3

Details on Quantifying the Effect of Procedural Differences

Table S3.1. Category counts for the BR dataset for scientific and grey literature included in the review.

(a) Nutrient recovery subset.

BR Coding Category	# of Studies TOTAL	# of Studies Scientific	# of Studies Grey	Fraction Scientific	Fraction Grey
URI + PREC-STRI	1	1	0	100	0
URI + SORP	4	4	0	100	0
URI + STRI	2	2	0	100	0
URI + XOTH	7	7	0	100	0
URI + MALG	3	3	0	100	0
URI + PREC	10	9	1	90	10
URI + MEMB	4	3	1	75	25
URI + PREC-BECS	2	1	1	50	50
URI + BECS	3	1	2	33	67
BLW + XOTH	1	1	0	100	0
BLW + BDEC-TDEC	1	1	0	100	0
BLW + BDEC-PREC	1	0	1	0	100
BLW + BTRT-PREC	1	0	1	0	100
BLW + PREC	1	0	1	0	100
SEW + BTRT-BECS	1	1	0	100	0
SEW + MALG-BDEC	1	1	0	100	0
SEW + MEMB-BDEC	1	1	0	100	0
SEW + MEMB-BECS	2	2	0	100	0
SEW + MEMB-PREC	1	1	0	100	0
SEW + MEMB-STRI	1	1	0	100	0
SEW + SORP-BDEC	1	1	0	100	0
SEW + SORP-MALG	2	2	0	100	0
SEW + SORP-PREC	2	2	0	100	0
SEW + TDEC-MALG	1	1	0	100	0
SEW + TDEC-SORP	2	2	0	100	0
SEW + BDEC-MALG	6	6	0	100	0
SEW + STRI	3	3	0	100	0
SEW + BECS	24	23	1	96	4
SEW + SORP	20	19	1	95	5
SEW + MALG	65	62	3	95	5
SEW + BTRT	18	15	3	83	17
SEW + PREC	28	25	3	89	11
SEW + TDEC	26	25	1	96	4
SEW + BDEC	50	45	5	90	10
SEW + MALG-TDEC	5	4	1	80	20
SEW + MALG–BDEC	11	8	3	73	27
SEW + XOTH	14	11	3	79	21
SEW + PROT	4	3	1	75	25
SEW + MEMB	3	2	1	67	33
SEW + BDEC-SORP	1	0	1	0	100
SEW + BTRT-SORP	1	0	1	0	100
SSA + PEXT	4	4	0	100	0
SSA + ASHT	3	3	0	100	0
OTH + BDEC	2	2	0	100	0
OTH + BECS	1	1	0	100	0
OTH + SORP	1	1	0	100	0
OTH + TDEC	1	1	0	100	0
GRW + SORP	1	0	1	0	100
**TOTAL**	**348**	**311**	**37**	**89**	**11**

(b) Nutrient reuse subset.

BR Coding Category	# of Studies TOTAL	# of Studies Scientific	# of Studies Grey	Fraction Scientific	Fraction Grey
URI @ urine	6	5	1	83	17
URI @ precipitate	2	1	1	50	50
BLW @ effluent	1	1	0	100	0
SEW @ effluent	51	48	3	94	6
SEW @ biosolid	39	38	1	97	3
SEW @ biochar	6	6	0	100	0
SEW @ sorbent	4	4	0	100	0
SEW @ protein	2	2	0	100	0
OTH @ effluent	4	4	0	100	0
OTH @ sorbent	1	1	0	100	0
OTH @ other	10	9	1	90	10
**TOTAL**	**126**	**119**	**7**	**94**	**6**

Table S3.2. Search modifications to the EW search string.

ELEMENTS

<Population>: WASH OR sanitation OR watsan OR ecosan OR toilet* OR latrine* OR urinal* OR urine OR yellowwater OR "yellow water" OR feces OR faeces OR brownwater OR "brown water" OR excreta OR excrement* OR "human waste" OR "human manure" OR humanure OR nightsoil OR "night soil" OR "night-soil" OR blackwater OR "black water" OR septage OR sewage OR sewerage OR wastewater OR "waste water" OR digestate* OR effluent* OR sludge OR biosolid*

<Intervention>: recover* OR *circul* OR reus* OR recycl* OR fertili* OR fertigat* OR conditioner* OR amendment* OR agricultur* OR "land application*"

<Outcome>: organic* OR nutrient* OR nitrogen OR urea OR ammonia OR ammonium OR phosphorus OR phosphorous OR phosphate OR phosphoric OR potassium OR potash OR fertili* OR *char OR *compost OR ash* OR biomass OR struvite OR vivianite OR worm*

BASELINE SEARCH

TITLE-ABS-KEY (<Population>)

AND

TITLE-ABS-KEY (<Intervention>)

AND

TITLE-ABS-KEY (<Outcome>)

AND NOT ORIG-LOAD-DATE > 20221231

TI : SEARCH IN TITLE FIELD ONLY

TITLE (<Population>)

AND

TITLE (<Intervention>)

AND

TITLE (<Outcome>)

AND NOT ORIG-LOAD-DATE > 20221231

MO : SEARCH WITH POPULATION TERM MODIFIERS

TITLE-ABS-KEY ((<Population>) W/2 (domestic OR sewage OR household OR households OR municipal OR municipality OR communal OR community OR city OR cities OR human OR urban OR rural OR urbanisation OR urbanization OR village OR villages OR source-separated OR source-separation OR "source separated" OR "source separation"))

AND

TITLE-ABS-KEY (<Intervention>)

AND

TITLE-ABS-KEY (<Outcome>)

AND NOT ORIG-LOAD-DATE > 20221231

OP : SEARCH WITH MORE RESTRICTIVE SEARCH OPERATOR

TITLE-ABS-KEY (<Population>)

AND

TITLE-ABS-KEY (<Intervention>)

W/3

TITLE-ABS-KEY (<Outcome>)

AND NOT ORIG-LOAD-DATE > 20221231

Table S3.3. Search queries of the replicated searches of previous reviews.

BR

TITLE-ABS-KEY(("organic carbon" OR DOC OR "organic C" OR "organic matter" OR nutrient* OR nitrogen OR nitrate OR nitrite OR ammoni* OR phosphorus OR phosphate) AND (wastewater OR "waste water" OR "storm water" OR stormwater OR blackwater OR "black water" OR greywater OR "grey water" OR graywater OR "gray water" OR sludge OR septage OR sewage OR "organic waste*" OR "septic sludge" OR sewerage* OR digestate* OR "toilet waste") AND (return OR recovery OR recover OR conversion OR convert OR circulate OR circular OR reuse OR recycle)) AND NOT ORIG-LOAD-DATE > 20231231

SA

#1

(TITLE-ABS-KEY ("urine separation" OR "urine treatment") OR TITLE-ABS-KEY ((domestic OR household OR municipal OR human) AND ("black*water" OR "brown*water" OR "yellow*water")) OR TITLE-ABS-KEY ("ecological sanitation" OR "eco*san" OR "eco sanitation") OR TITLE ((waste*water OR sanitation OR sludge) AND (recover* OR recycl*) AND (*nutrient OR nitrogen OR potassium OR phosphorus)) OR TITLE ((sanitation OR waste*water) AND (farming OR agriculture* OR horticultur*)) AND TITLE-ABS-KEY((domestic OR household OR municipal OR human))) AND NOT ORIG-LOAD-DATE > 20231231

#2

TITLE ((sanitation OR urine OR yellow*water OR faeces OR "faecal matter" OR brown*water OR excreta OR black*water OR waste*water OR sewage OR sludge OR biosolid*) AND (*nutrient* OR phosph* OR nitrogen OR ammoni* OR potas* OR farming OR agricultur* OR horticultur*) AND (recover* OR recycle* OR re*use OR valorisation OR farming OR agricultur* OR horticultur*)) AND NOT ORIG-LOAD-DATE > 20231231

UM

#1

(TITLE-ABS-KEY(urine) AND ALL((recover*)) AND ALL((nutrient*))) AND NOT ORIG-LOAD-DATE > 20231231

#2

TITLE-ABS-KEY (((urine OR yellowwater OR "yellow water") AND (recover* OR circul* OR recycl*) AND (nutrient* OR nitrogen OR urea OR ammonia OR ammonium OR phosphorus OR phosphate OR potassium OR fertili* OR struvite))) AND NOT ORIG-LOAD-DATE > 20231231

EW

TITLE-ABS-KEY (WASH OR sanitation OR watsan OR ecosan OR toilet* OR latrine* OR urinal*) OR TITLE-ABS-KEY (urine OR yellowwater OR "yellow water") OR TITLE-ABS-KEY (feces OR faeces OR brownwater OR "brown water") OR TITLE-ABS-KEY (excreta OR excrement* OR "human waste" OR "human manure" OR humanure OR nightsoil OR "night soil" OR "night-soil" OR blackwater OR "black water") OR TITLE-ABS-KEY (septage OR sewage OR sewerage OR wastewater OR "waste water" OR digestate* OR effluent* OR sludge OR biosolid*) AND TITLE-ABS-KEY (recover* OR *circul* OR reus* OR recycl* OR fertili* OR fertigat* OR conditioner* OR amendment* OR agricultur* OR "land application*") AND TITLE-ABS-KEY (organic* OR nutrient* OR nitrogen OR urea OR ammonia OR ammonium OR phosphorus OR phosphorous OR phosphate OR phosphoric OR potassium OR potash OR fertili* OR *char OR *compost OR ash* OR biomass OR struvite OR vivianite OR worm*) AND NOT ORIG-LOAD-DATE > 20231231

Table S3.4. Search queries of the modified EW searches.

EW

TITLE-ABS-KEY (WASH OR sanitation OR watsan OR ecosan OR toilet* OR latrine* OR urinal* OR urine OR yellowwater OR "yellow water" OR feces OR faeces OR brownwater OR "brown water" OR excreta OR excrement* OR "human waste" OR "human manure" OR humanure OR nightsoil OR "night soil" OR "night-soil" OR blackwater OR "black water" OR septage OR sewage OR sewerage OR wastewater OR "waste water" OR digestate* OR effluent* OR sludge OR biosolid*) AND TITLE-ABS-KEY (recover* OR *circul* OR reus* OR recycl* OR fertili* OR fertigat* OR conditioner* OR amendment* OR agricultur* OR "land application*") AND TITLE-ABS-KEY (organic* OR nutrient* OR nitrogen OR urea OR ammonia OR ammonium OR phosphorus OR phosphorous OR phosphate OR phosphoric OR potassium OR potash OR fertili* OR *char OR *compost OR ash* OR biomass OR struvite OR vivianite OR worm*) AND NOT ORIG-LOAD-DATE > 20221231

EW-MOD1

TITLE (WASH OR sanitation OR watsan OR ecosan OR toilet* OR latrine* OR urinal*) OR TITLE (urine OR yellowwater OR "yellow water") OR TITLE (feces OR faeces OR brownwater OR "brown water") OR TITLE (excreta OR excrement* OR "human waste" OR "human manure" OR humanure OR nightsoil OR "night soil" OR "night-soil" OR blackwater OR "black water") OR TITLE (septage OR sewage OR sewerage OR wastewater OR "waste water" OR digestate* OR effluent* OR sludge OR biosolid*) AND TITLE (recover* OR *circul* OR reus* OR recycl* OR fertili* OR fertigat* OR conditioner* OR amendment* OR agricultur* OR "land application*") AND TITLE (organic* OR nutrient* OR nitrogen OR urea OR ammonia OR ammonium OR phosphorus OR phosphorous OR phosphate OR phosphoric OR potassium OR potash OR fertili* OR *char OR *compost OR ash* OR biomass OR struvite OR vivianite OR worm*) AND NOT ORIG-LOAD-DATE > 20221231

EW-MOD2

TITLE-ABS-KEY ((WASH OR sanitation OR watsan OR ecosan OR toilet* OR latrine* OR urinal* OR urine OR yellowwater OR "yellow water" OR feces OR faeces OR brownwater OR "brown water" OR excreta OR excrement* OR "human waste" OR "human manure" OR humanure OR nightsoil OR "night soil" OR "night-soil" OR blackwater OR "black water" OR septage OR sewage OR sewerage OR wastewater OR "waste water" OR digestate* OR effluent* OR sludge OR biosolid*) W/2 (domestic OR sewage OR household OR households OR municipal OR municipality OR communal OR community OR city OR cities OR human OR urban OR rural OR urbanisation OR urbanization OR village OR villages OR source-separated OR source-separation OR "source separated" OR "source separation")) AND TITLE-ABS-KEY (recover* OR *circul* OR reus* OR recycl* OR fertili* OR fertigat* OR conditioner* OR amendment* OR agricultur* OR "land application*") AND TITLE-ABS-KEY (organic* OR nutrient* OR nitrogen OR urea OR ammonia OR ammonium OR phosphorus OR phosphorous OR phosphate OR phosphoric OR potassium OR potash OR fertili* OR *char OR *compost OR ash* OR biomass OR struvite OR vivianite OR worm*) AND NOT ORIG-LOAD-DATE > 20221231

EW-MOD3

TITLE-ABS-KEY ((WASH OR sanitation OR watsan OR ecosan OR toilet* OR latrine* OR urinal* OR urine OR yellowwater OR "yellow water" OR feces OR faeces OR brownwater OR "brown water" OR excreta OR excrement* OR "human waste" OR "human manure" OR humanure OR nightsoil OR "night soil" OR "night-soil" OR blackwater OR "black water" OR septage OR sewage OR sewerage OR wastewater OR "waste water" OR digestate* OR effluent* OR sludge OR biosolid*)) AND TITLE-ABS-KEY ((recover* OR *circul* OR reus* OR recycl* OR fertili* OR fertigat* OR conditioner* OR amendment* OR agricultur* OR "land application*") W/3 (organic* OR nutrient* OR nitrogen OR urea OR ammonia OR ammonium OR phosphorus OR phosphorous OR phosphate OR phosphoric OR potassium OR potash OR fertili* OR *char OR *compost OR ash* OR biomass OR struvite OR vivianite OR worm*)) AND NOT ORIG-LOAD-DATE > 20221231

Table S3.5. Search queries of the original searches in previous reviews

BR

TITLE-ABS-KEY(("organic carbon" OR DOC OR "organic C" OR "organic matter" OR nutrient* OR nitrogen OR nitrate OR nitrite OR ammoni* OR phosphorus OR phosphate) AND (wastewater OR "waste water" OR "storm water" OR stormwater OR blackwater OR "black water" OR greywater OR "grey water" OR graywater OR "gray water" OR sludge OR septage OR sewage OR "organic waste*" OR "septic sludge" OR sewerage* OR digestate* OR "toilet waste") AND (return OR recovery OR recover OR conversion OR convert OR circulate OR circular OR reuse OR recycle)) AND (LIMIT-TO (PUBYEAR,2017) OR LIMIT-TO (PUBYEAR,2016) OR LIMIT-TO (PUBYEAR,2015) OR LIMIT-TO (PUBYEAR,2014) OR LIMIT-TO (PUBYEAR,2013))

SA

1

TITLE-ABS-KEY ("urine separation" OR "urine treatment") ORIG-LOAD-DATE < 20170201

TITLE-ABS-KEY ((domestic OR household OR municipal OR human) AND ("black*water" OR "brown*water" OR "yellow*water")) AND ORIG-LOAD-DATE < 20170201

TITLE-ABS-KEY (("ecological sanitation" OR "eco*san" OR "eco sanitation")) AND ORIG-LOAD-DATE < 20170201

TITLE ((waste*water OR sanitation OR sludge) AND (recover* OR recycl*) AND (*nutrient OR nitrogen OR potassium OR phosphorus)) AND ORIG-LOAD-DATE < 20170201

TITLE ((sanitation OR waste*water) AND (farming OR agriculture* OR horticultur*)) AND TITLE-ABS-KEY((domestic OR household OR municipal OR human)) AND ORIG-LOAD-DATE < 20170201

#2

(TITLE ((sanitation OR urine OR yellow*water OR faeces OR "faecal matter" OR brown*water OR excreta OR black*water OR waste*water OR sewage OR sludge OR biosolid*)) AND TITLE ((*nutrient* OR phosph* OR nitrogen OR ammoni* OR potas* OR farming OR agricultur* OR horticultur*)) AND TITLE ((recover* OR recycl* OR re*use OR valorisation OR farming OR agricultur* OR horticultur*))) AND NOT TITLE (animal OR livestock OR herbivore OR poultry OR swine OR pig* OR bovine OR cow* OR cattle OR dairy OR milk OR meat OR manure) AND NOT TITLE (hospital OR kidney OR blood OR child* OR women OR men OR schistosomiasis) AND NOT TITLE (oil* OR petroleum OR coal OR mining OR mine OR paper* OR rubber OR pulp* OR *mill OR tannery OR beamhouse OR brewery OR dredg* OR concrete OR coking OR semiconductor OR electroplating OR pharmaceutic* OR salin* OR hydroxide OR *ethanol OR furnace OR silo* OR printing OR "disposable diapers" OR manufactur* OR sugarbeet OR potato OR "dental metal") AND NOT TITLE (risk OR toxic* OR disease OR health OR occupational* OR work* OR virologic* OR bacteriologic* OR hygien* OR epidemiolog* OR infection OR reservoir OR hydrogeologic OR kinetic* OR speciation OR uptake OR dissipation OR morphology OR fate OR mobility OR determination OR metabolite OR exposure OR prevalence OR monitoring OR hazard OR runoff OR bloom*) AND NOT TITLE (impact* OR planning OR evaluation OR assess* OR model* OR effect OR policy OR consequence* OR attitude OR perce* OR preference*) AND NOT TITLE ((industr*) AND NOT (municipal OR commun* OR domestic OR city OR urban OR campus OR regional)) AND NOT TITLE ((pollutant* OR contamina* OR pah OR las OR "linear alkylbenzene sulfonate" OR phenol* OR pae OR pcb OR dioxin OR furan OR polybrom* OR perfluor* OR "heavy metal*" OR pathogen* OR virus OR bacteri* OR helminth* OR giardi* OR salmonella OR *samine OR estrogen OR pcdd or antibiotic* or pesticide* OR aromatic OR hydrocarbon* OR triclo* OR surfactant* OR ibuprofen OR estrone OR estradiol OR phthalate OR drug* OR glyphosate) AND NOT (remov* OR inactivat* OR destr*)) AND NOT TITLE ("*cult* waste*" OR "*farming waste*" OR "shrimp waste*" OR "fish waste*" OR "household garbage" OR "phosph* sludge" OR "phosphoric acid sludge" OR "alum* sludge") AND NOT TITLE((social OR economic) AND NOT environmental)

UM

#1

(TITLE-ABS-KEY(urine) AND ALL((recover*)) AND ALL((nutrient*))) AND PUBYEAR < 2022

#2

TITLE-ABS-KEY (((urine OR yellowwater OR "yellow water") AND (recover* OR circul* OR recycl*) AND (nutrient* OR nitrogen OR urea OR ammonia OR ammonium OR phosphorus OR phosphate OR potassium OR fertili* OR struvite))) AND PUBYEAR < 2022

#3

(TITLE-ABS-KEY ((urine OR urinal OR yellowwater OR "yellow water" OR "yellow-water") AND (recover* OR *circul* OR reus* OR recycl* OR fertili* OR fertigat* OR conditioner* OR amendment* OR agricultur* OR "land application*") AND (organic* OR nutrient* OR biosolid OR nitrogen OR urea OR ammonia OR ammonium OR phosphorus OR phosphate OR phosphoric OR potassium OR potash OR fertili* OR *char OR *compost OR ash* OR biomass OR struvite OR vivianite OR worm*))) AND PUBYEAR < 2022
