# Supplementary material for: Mapping large bodies of research in environmental sciences: insights from compiling evidence on the recovery and reuse of nutrients found in human excreta and domestic wastewater
Source: Environ Evid. 2025 Jul 14;14:13. doi: 10.1186/s13750-025-00366-5 (PMC12261714; doi:10.1186/s13750-025-00366-5)
Supplement: Supplementary file 4 — Additional file 4: Opportunities for Efficiency Gains [file 13750_2025_366_MOESM4_ESM.pdf]

ENVIRONMENTAL EVIDENCE

Mapping Large Bodies of Research in Environmental Sciences: Insights from Compiling Evidence on the Recovery and Reuse of Nutrients Found in Human Excreta and Domestic Wastewater

Robin Harder

https://doi.org/10.1186/s13750-025-00366-5

SUPPORTING INFORMATION 4

Opportunities for Efficiency Gains

Bibliographic APIs

Given limitations on how many search results that can be exported at once from web-based user interfaces of bibliographic databases, exporting search results becomes tedious if the search has many hits – even more so if multiple databases are searched. Application programming interfaces (APIs) are a type of software interface that use a set of definitions and protocols to offer a service to other pieces of software. Bibliographic APIs are available for a large number of bibliographic data sources and provide a standardized way of retrieving bibliographic data from these sources through a software interface rather than manually through a website (Scalfani et al., 2023; Harder, 2024; Atkinson, 2025). One of the key advantages of bibliographic APIs is that they bypass the manual steps involved in searching and retrieving bibliographic data through a website. Bibliographic APIs thus allow to swiftly run a large number of distinct searches and automatically save the respective search hits for further processing.

Data Storage

Handling large amounts of data in spreadsheets is less than ideal. Database management systems offer a superior alternative to store data in a structured way. With the help of versatile commands offered by standard languages for database creation and manipulation, the data can then be cleaned and processes in targeted and efficient ways. One of the key advantages of using a database management system is that data operations can be executed not only manually through a user interface but also with the help of customized tools and scripts. The Egestabase online evidence platform is stored in a relational database (MySQL). While relational databases offer considerable advantages over flat data tables (such as spreadsheets), the downside is that they require a detailed level of prior knowledge regarding the structure of the evidence and the applications of the database (Wolffe et al., 2020) – which indeed caused some headache during the development of Egestabase (the structure of the database had to be changed multiple times). Wolffe and colleagues (2020) convincingly argue that schemaless data storage, for example in knowledge graphs, would be even better than a relational database and should in fact be the first choice when it comes to data storage for systematic maps.

Bespoke Screening and Coding Tool

Established online tools designed to facilitate literature reviews may not be optimized for handling large numbers of studies – I experienced handling any more than a few hundred or perhaps a few thousand records on for instance EPPI Reviewer as neither particularly practical nor sufficiently efficient. For large maps, dedicated online tools such as EPPI Reviewer may thus become increasingly impractical and inefficient for screening and coding. To allow for a more time efficient screening and coding process, I developed a web-based bespoke screening and coding tool (written in PHP and directly connected to the MySQL database). While this bespoke tool meant a sizeable initial effort, the screening and coding process became at least ten times faster than it would have been using EPPI Reviewer. There is one caveat, however. This bespoke tool turned out to be so specific that training other people to use it was not trivial (it actually failed miserably). While not impossible, it would appear that building a bespoke tool that can easily be operated by multiple reviewers is much more difficult and time consuming than building a tool that is only going to be used by its creator. At the same time, there is a risk that some of the key features that boost time-efficiency may be less suited for a multi-reviewer implementation.

Efficient Deduplication

Searching multiple databases in addition necessitates deduplication. Wherever a DOI is not available, deduplication becomes a fuzzy business that may require a lot of manual work or a dedicated script with some degree of manual checking. But even with a fair bit of scripting, deduplication may become rather tiring and swallow up resources that may later lack elsewhere in the process. A more efficient deduplication than what I managed to implement when compiling Egestabase would be desirable. I am pretty sure that good solutions exist somewhere 'out there' – so watch out for them.

Efficient Filtering

If screening and coding is done randomly, reviewers constantly need to mentally switch between excludes and includes and across distinct topics within the includes – which is tiring and inefficient. This issue can be mitigated by splitting studies into more homogeneous subsets prior to screening and coding. One way to achieve this is by filtering based on specific terms. For instance, terms from the medical field can be used to identify studies that should be excluded whereas specific combinations of terms that constitute the original search string can be used to filter studies that belong to a certain subdomain. While the benefits of filtering are obvious, it is perhaps less obvious that it also matters how the filter is implemented. If changing filter settings requires several clicks and the process of applying and running the filter takes up to 30 seconds, this is rather annoying and quickly amounts to a substantial chunk of time wasted. A filter that can be set with one click and that takes less than a second to run would be much better. Relatedly, it helps if integration of Boolean operators (i.e., AND, OR, NOT) is straightforward. One may for instance want to filter out studies that feature the term wastewater along with terms that indicate industrial wastewater but not terms for domestic wastewater. Ideally, this should not take more than a few seconds. In fact, the bespoke screening and coding tool described above was so much faster not least because setting and running filters was very fast.

Efficient Prepopulation of Screening and Coding Fields

The benefit of efficient filtering can be further amplified if combined with prepopulated screening and coding fields in the screening and coding tool. The reviewer then simply needs to browse through the filter results and adjust the prepopulated fields only where needed (if the filter works well, only a few adjustments should be needed). While the idea of prepopulating screening and coding fields is not particularly novel or innovative in itself, also here the implementation matters. Ideally, prepopulation settings can be changed and applied in a matter of a few seconds.

Parsimonious Coding Scheme

While there are many possible coding schemes that are both logical and consistent, some of them may necessitate that reviewers dig deeper into the studies to extract the required information. For example, wastewater irrigation may refer to the irrigation with raw wastewater or to the irrigation with treated wastewater effluents. In the absence of convincing reasons why these two subdomains should be distinguished, it might be a good idea to subsume them into a single coding category. In the case of the Egestabase mapping process, this meant that over 3 200 studies could be classified pretty much based on title and abstract without the need to retrieve and sift through the full texts. In contrast, the distinction of membrane separation for water extraction and membrane separation for nutrient extraction was deemed desirable – and feasible as it concerned less than 300 studies of which many held sufficient information for this distinction in the title and abstract.

Full Text Retrieval

Retrieving full texts is another tedious task. Full text retrieval appears to be way more cumbersome than necessary and retrieving thousands of them one by one certainly is not a viable option. At the same time, it would appear that publishers and shadow libraries (such as Sci-Hub) alike go to great lengths to make sure that full texts cannot be retrieved in an automatized way – which makes total sense from their perspective. Yet from the perspective of a researcher, it really hampers the research process. Even though I found ways to retrieve about 80 percent of the relevant PDFs in a semi-automatized way, the process of retrieving the PDFs still was more convoluted and time consuming than it should. Ideally, one would be able to automatically retrieve large numbers of full texts and download PDFs based on a list of DOIs. Unfortunately, I did not have this functionality at hand when compiling Egestabase. But I am aware that efforts seem to be under way in the evidence synthesis community – so watch out for them.

Machine Learning

Machine learning algorithms are a promising way to streamline screening and coding by means of providing automated (preliminary or final) screening and coding decisions. EPPI Reviewer in fact features a number of such algorithms. While these algorithms have proved to be very helpful for smaller reviews, for the rather large datasets I have worked with, their performance unfortunately appeared to be rather disappointing, both in terms of accuracy and speed. Machine learning was thus not applied when compiling Egestabase – mainly because nobody on the team had the skills needed to do this in a customized way. This said, I strongly believe that machine learning in principle can be successfully applied to streamline the mapping of large bodies of literature – provided that algorithms are more customizable and targeted to a specific domain than what can reasonably be implemented on a tool like EPPI Reviewer that has to work across all kinds of research domains. Text mining for instance seems to be an approach that holds considerably promise (O'Mara-Eves et al., 2015). Getting somebody on the team who really masters machine learning thus may be a good idea.

References

Atkinson, C.F., 2025. AI-pocalypse now: Automating the systematic literature review with SPARK (Systematic processing and automated review Kit) – gathering, organising, filtering, and scaffolding. MethodsX 14, 103129. https://doi.org/10.1016/j.mex.2024.103129

Harder, R., 2024. Using Scopus and OpenAlex APIs to retrieve bibliographic data for evidence synthesis. A procedure based on Bash and SQL. MethodsX 12, 102601. https://doi.org/10.1016/j.mex.2024.102601

O'Mara-Eves, A., Thomas, J., McNaught, J., Miwa, M., Ananiadou, S., 2015. Using text mining for study identification in systematic reviews: a systematic review of current approaches. Syst Rev 4, 5. https://doi.org/10.1186/2046-4053-4-5

Scalfani, V.F., Walker, K.W., Simpson, L., Fernandez, A.M., Patel, V.D., Ramig, A., Gomes, C., Moen, M.T., Nguyen, A.M., 2023. Creating a Scholarly API Cookbook: Supporting Library Users with Programmatic Access to Information. ISTL. https://doi.org/10.29173/istl2766

Wolffe, T.A.M., Vidler, J., Halsall, C., Hunt, N., Whaley, P., 2020. A Survey of Systematic Evidence Mapping Practice and the Case for Knowledge Graphs in Environmental Health and Toxicology. Toxicological Sciences 175, 35–49. https://doi.org/10.1093/toxsci/kfaa025
